# Supplementary figures and images for: Defining morphologically and genetically distinct GABAergic/cholinergic amacrine cell subtypes in the vertebrate retina
Source: PLoS Biol. 2024 Feb 16;22(2):e3002506. doi: 10.1371/journal.pbio.3002506 (PMC10914270; doi:10.1371/journal.pbio.3002506)

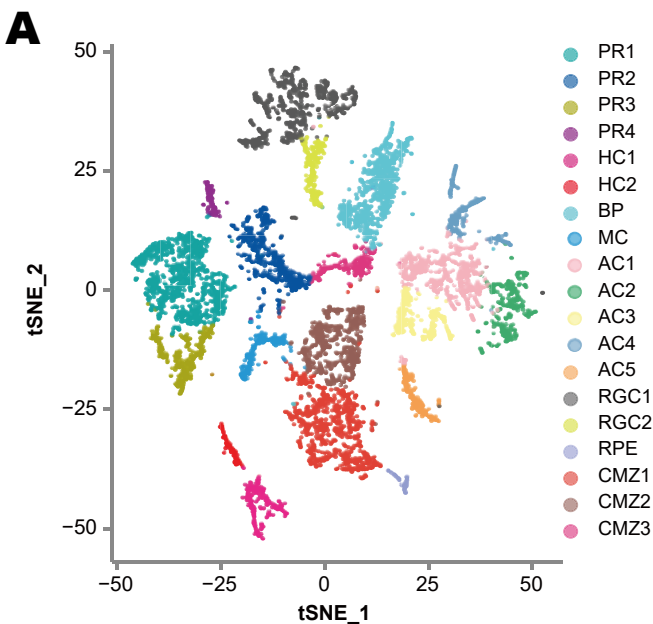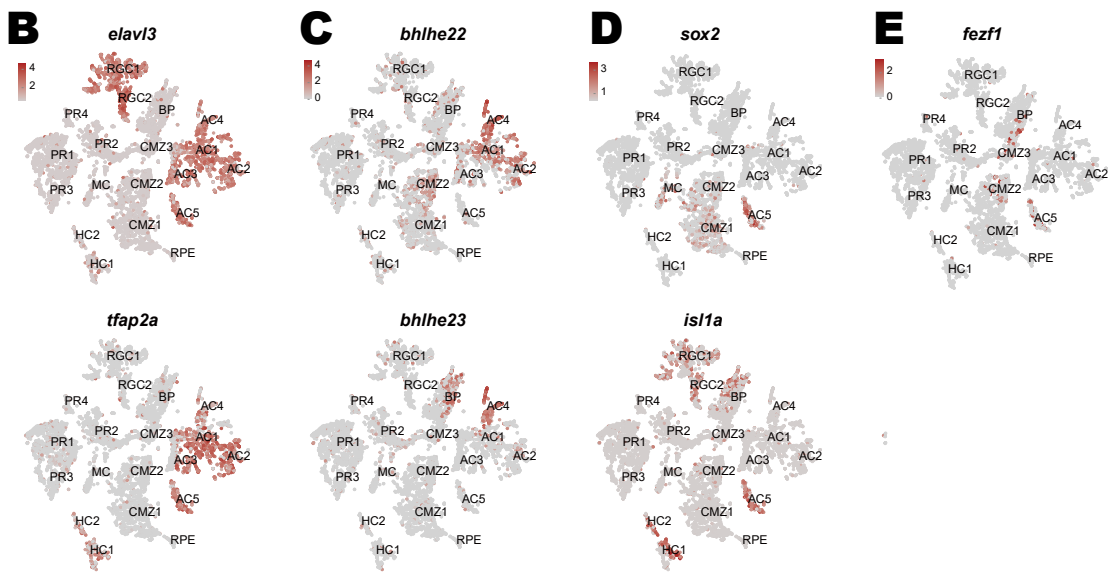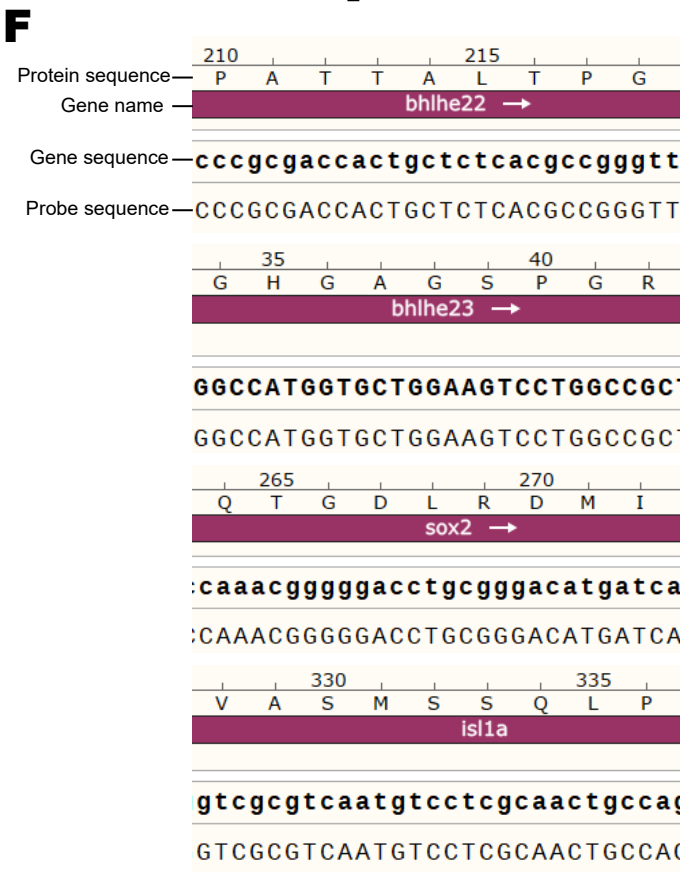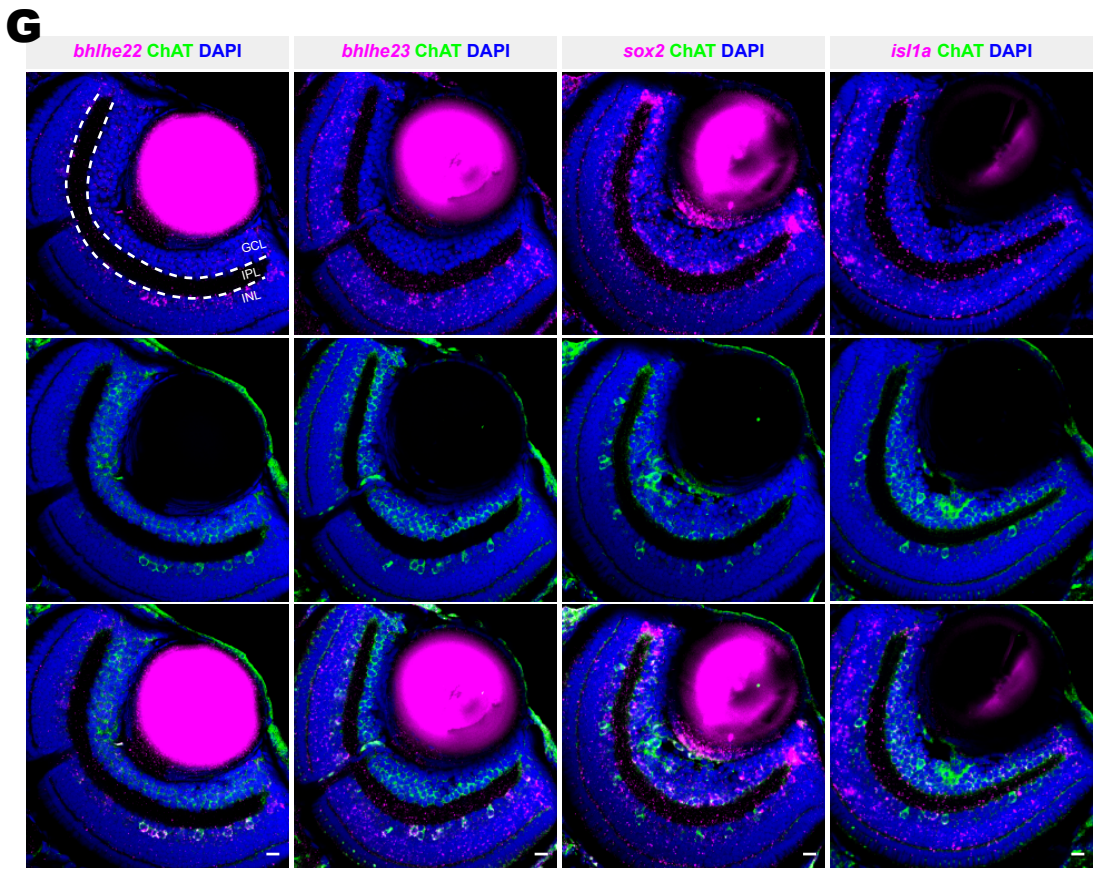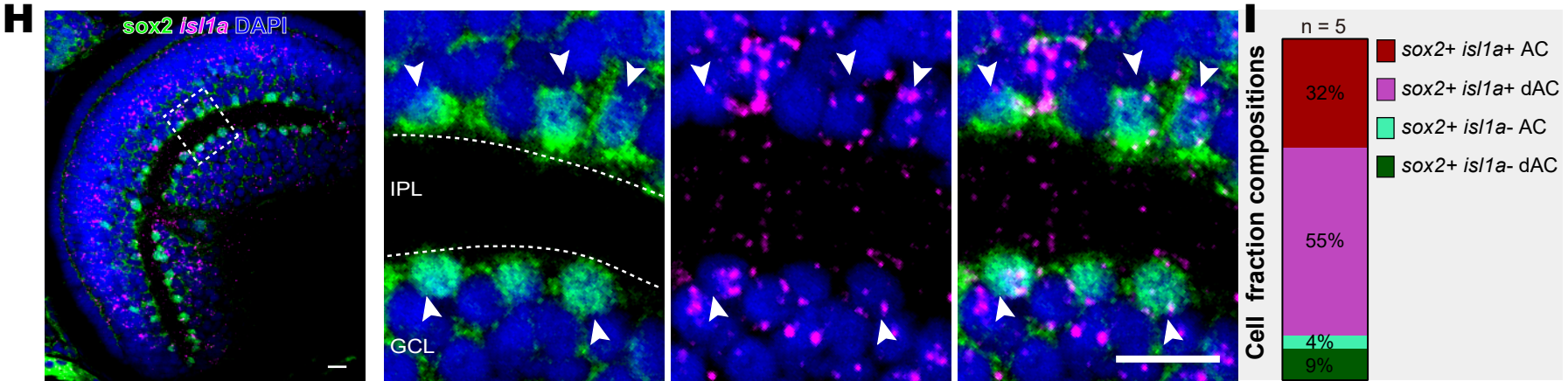

Supplement: S1 Fig — (A) t-SNE plot showing 19 clusters of the zebrafish retina (72 hpf). Each cluster is identified as one cell type according to highly expressed cluster-specific marker genes. (B) t-SNE feature plot showing elval3 and tfap2a expression level in 19 clusters of the zebrafish retina. (C) t-SNE feature plot showing bhlhe22 and bhlhe23 expression level. (D) t-SNE feature plot showing sox2 and isl1a expression level. (E) t-SNE feature plot showing fezf1 and tenm3 expression level. (F) The validation of riboprobe sequences for marker TFs of cluster AC4 (bhlhe22 and bhlhe23) and AC5 (sox2 and isl1a). (G) Expression patterns of riboprobes in (F) using in situ hybridization (magenta) combined with ChAT immunostaining (green) of the 5-dpf zebrafish. (H) Validation of cluster AC5 marker isl1a (magenta) using in situ hybridization combined with SOX2 (green) immunostaining. (I) Cell fraction composition analysis of sox2+ cells in (H). Solid white arrow head indicated sox2+ isl1a+ cells. The data underlying this figure can be found in S3 Data. Scale bars, 10 μm. AC, amacrine cell; ChAT, choline acetyltransferase; dAC, displaced amacrine cell; dpf, days post-fertilization; GCL, ganglion cell layer; IPL, inner plexiform layer; TF, transcription factor. (PDF) [file pbio.3002506.s001.pdf]

**A**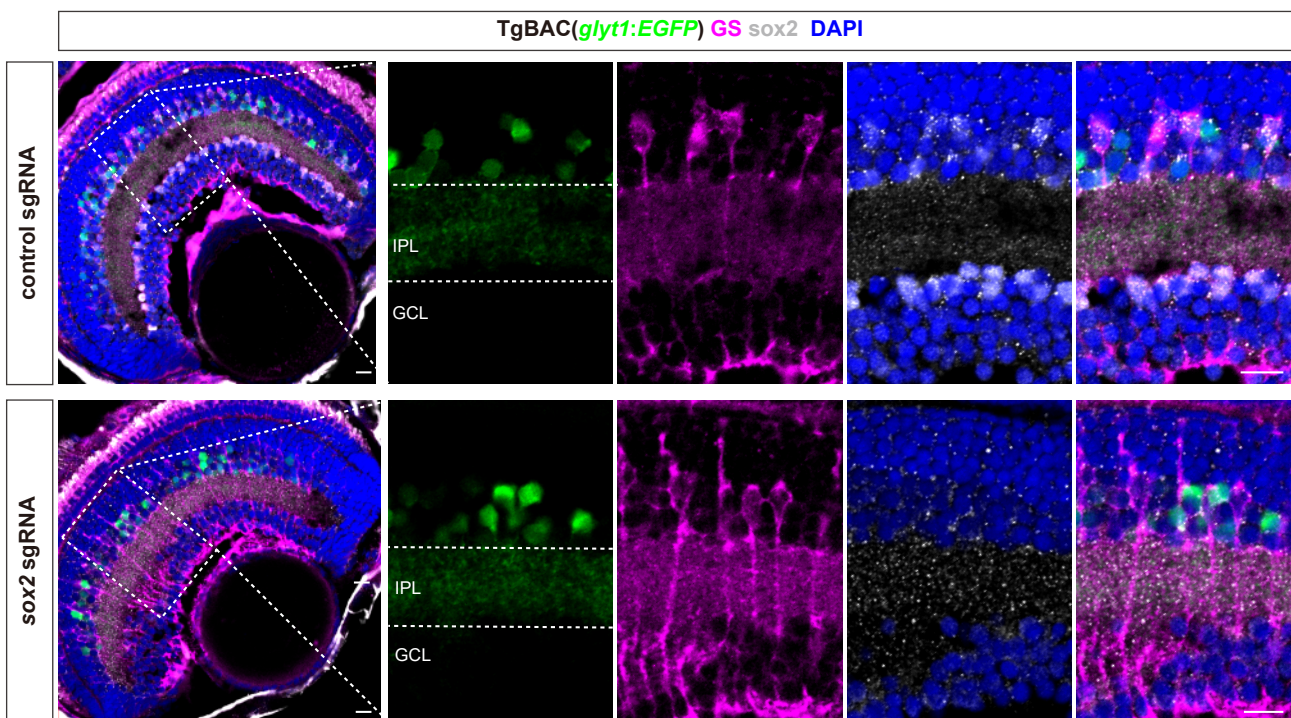**B**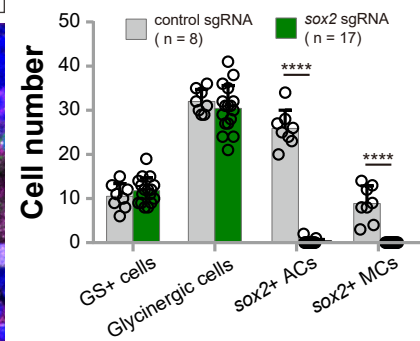**C**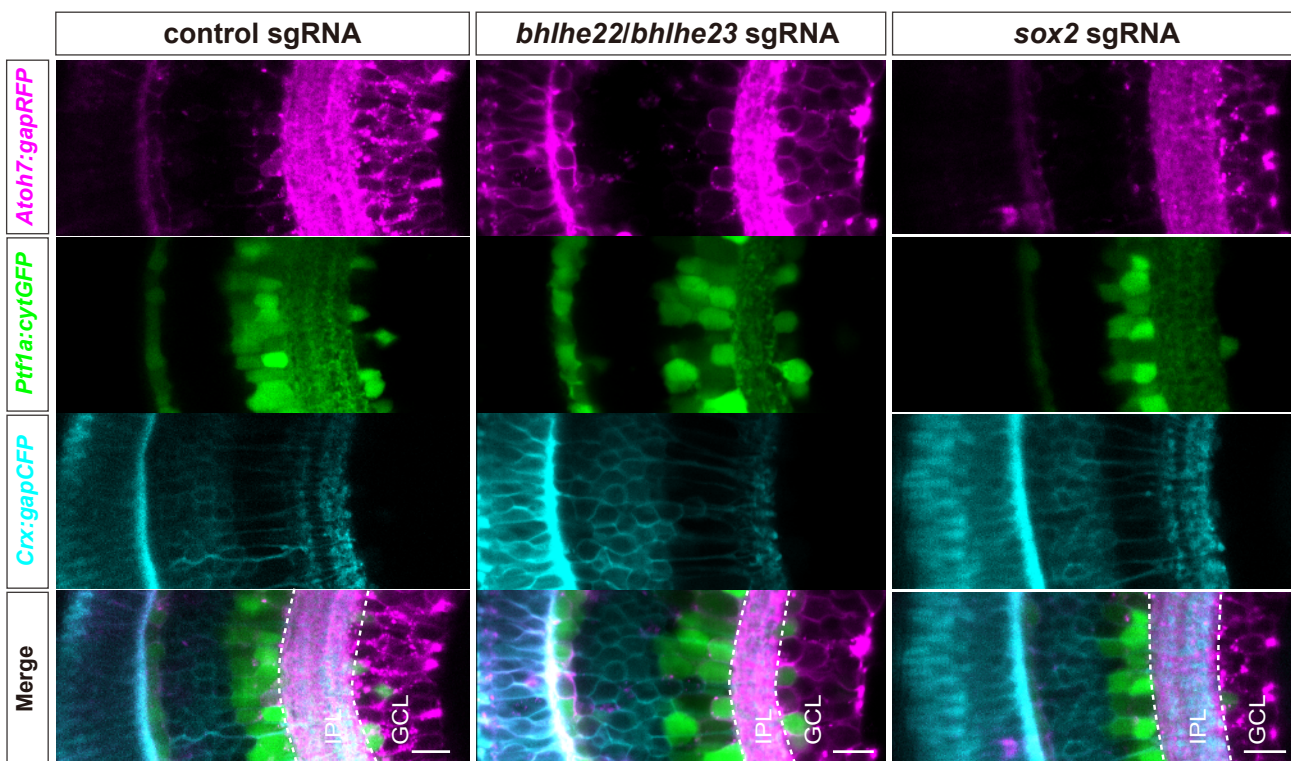**D**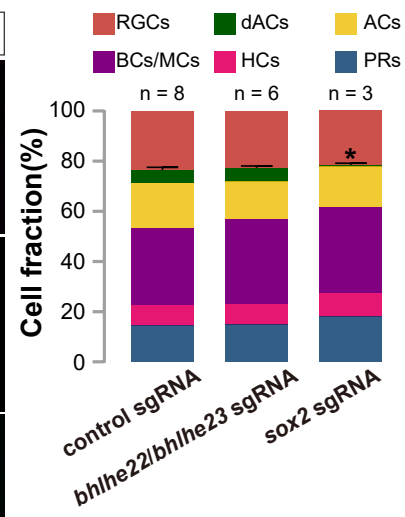

Supplement: S3 Fig — (A) Representative images showing patterns of sox2+ ACs, sox2+ MCs and GS+ MCs after disruption of sox2 in TgBAC(glyt1: EGFP). (B) Quantification in (A). (C) Representative images showing Sofa1 fish (generated by crossing Atoh7: gapRFP, Ptf1a: cytGFP, and Crx:gapCFP) patterns after disruption of sox2 and bhlhe22/bhlhe23. (D) Cell fraction composition of major retinal cell types of 3 groups in (C). The data underlying this figure can be found in S3 Data. Data are presented as mean ± SD, Mann–Whitney test. * 0.01 < = p <0.05,**** p <0.0001. Scale bars, 10 μm. AC, amacrine cell; BC, bipolar cell; dAC, displaced amacrine cell; GCL, ganglion cell layer; HC, horizontal cell; IPL, inner plexiform layer; MC, müller cell; RGC, retinal ganglion cell; PR, photoreceptor cell; sgRNA, small guide RNA. (PDF) [file pbio.3002506.s003.pdf]

**Fig .S5**

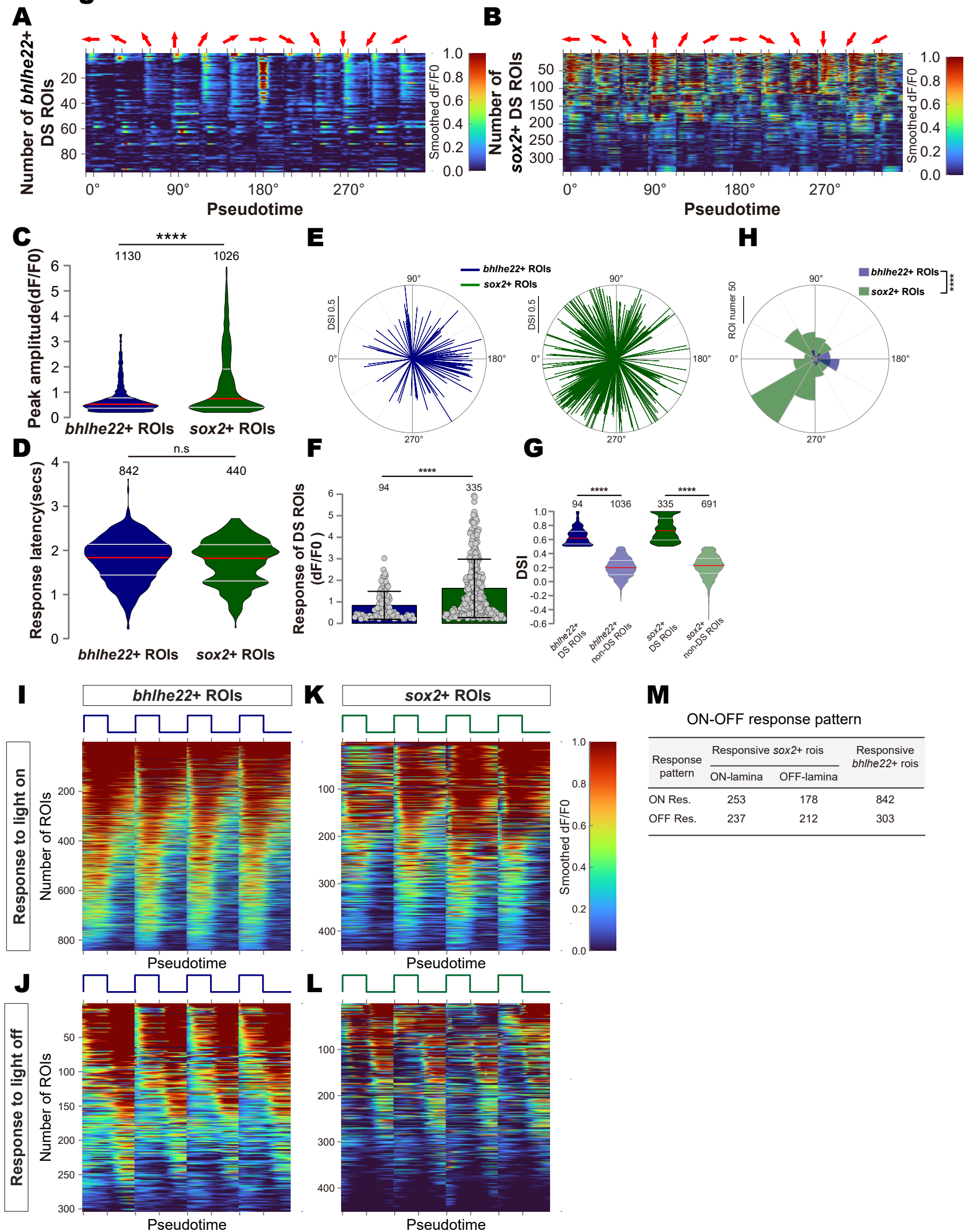

Supplement: S5 Fig — (A) Heatmap showing the response of all DS bhlhe22+ ROIs in (Fig 5C). (B) Heatmap showing the response of all DS sox2+ ROIs in (Fig 5G). (C) Violin plot showing peak amplitude of sox2+ and bhlhe22+ responsive ROIs to moving bar. The gray lines indicate the quartiles, and the red lines indicate the median. Mann–Whitney test. **** p <0.0001. (D) Violin plot showing response latency of light on response of sox2+ and bhlhe22+ responsive trials. Mann–Whitney test. (E) Polar plot of distribution of DSI and PD of bhlhe22+ and sox2+ DS ROIs. Scale bar, DSI value = 0.5. (F) Bar plot of responses of DS bhlhe22+ and sox2+ ROIs in Fig 5C and 5G. Data are shown in mean ± SD. Mann–Whitney test. (G) Violin plot showing DSI of DS and non-DS ROIs in Fig 5C and 5G. Mann–Whitney test. (H) Histogram polar plot of PD distribution of DS ROIs of 2 types of cells. Scale bar, ROI number = 50. Cumulative frequency comparison with Kolmogorov–Smirnov test. (I and J) Heatmap of response to light on (I, ON Res.) and off (J, OFF Res.) of bhlhe22+ ROIs. The schematic showing visual stimuli paradigm of full-field screen spot was on top of heatmap. (K and L) Heatmap of response to light on (K, ON Res.) and off (L, OFF Res.) of sox2+ ROIs. (M) Statistics of response patterns in (I to L). The data underlying this figure can be found in S3 Data. DS, direction selective; DSI, direction selectivity index; PD, preferential direction; ROI, region of interest. (PDF) [file pbio.3002506.s005.pdf]

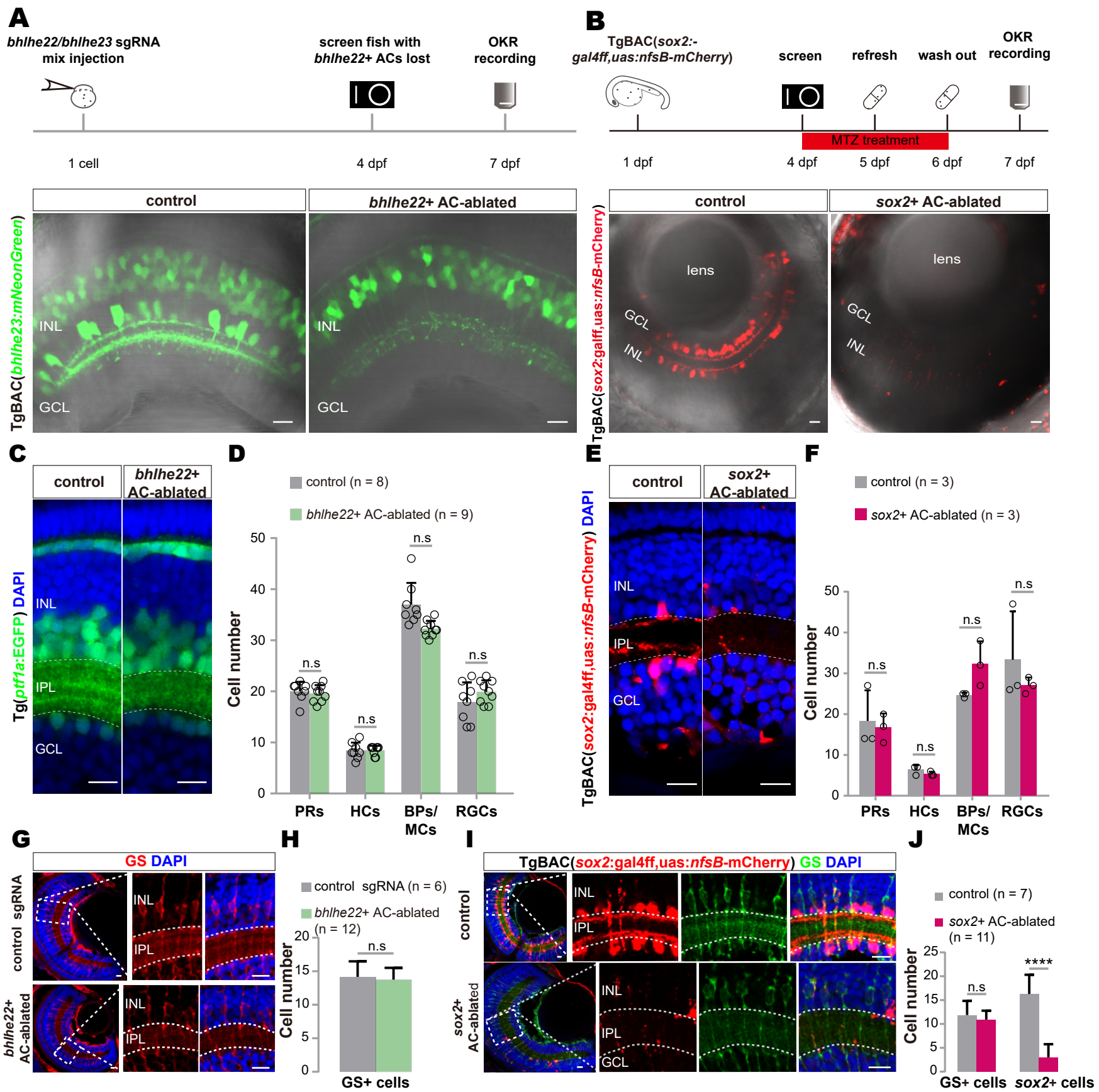

Supplement: S6 Fig — (A) Workflow(upper) and representative images (bottom) showing the OKR assay of bhlhe22+ ACs-ablated zebrafish. (B) Workflow (upper) and representative images (bottom) showing the OKR assay of sox2+ AC-ablated zebrafish. (C and D) Representative image (C) and quantification (D) of a section (30 × 100 μm) of whole-mount Tg(ptf1a: EGFP) retina with bhlhe22/bhlhe23 codisruption to ablate bhlhe22+ ACs. Cell number and types were defined by cell body location in layers. (E and F) Representative image (E) and quantification (F) a frozen section of (30 × 100 μm) TgBAC(sox2: gal4ff, uas:nfsB-mCherry) retina with MTZ to ablate sox2+ ACs. Cell number and types were defined by cell body location in layers. (G and H) Representative image labeling with MCs marker anti-GS (G) and quantification (H) of a frozen section of (100 × 100 μm) wild-type with bhlhe22/bhlhe23 codisruption to ablate bhlhe22+ ACs. (I and J) Representative image (I) and quantification (J) of a frozen section of (100 × 100 μm) TgBAC(sox2:gal4ff,uas:nfsB-mCherry) labeling with GS after MTZ treatment. The data underlying this figure can be found in S3 Data. Data are collected from 5 to 8 dpf the larval zebrafish, presented as mean ± SD, Mann–Whitney test. **** p <0.0001. Scale bars, 10 μm. AC, amacrine cell; BP, bipolar cell; GCL, ganglion cell layer; HC, horizontal cell; INL, inner nuclear layer; IPL, inner plexiform layer; MC, müller cell; MTZ, metronidazole; OKR, optokinetic reflex; PR, photoreceptor cell; RGC, retinal ganglion cell; sgRNA, small guide RNA. (PDF) [file pbio.3002506.s006.pdf]

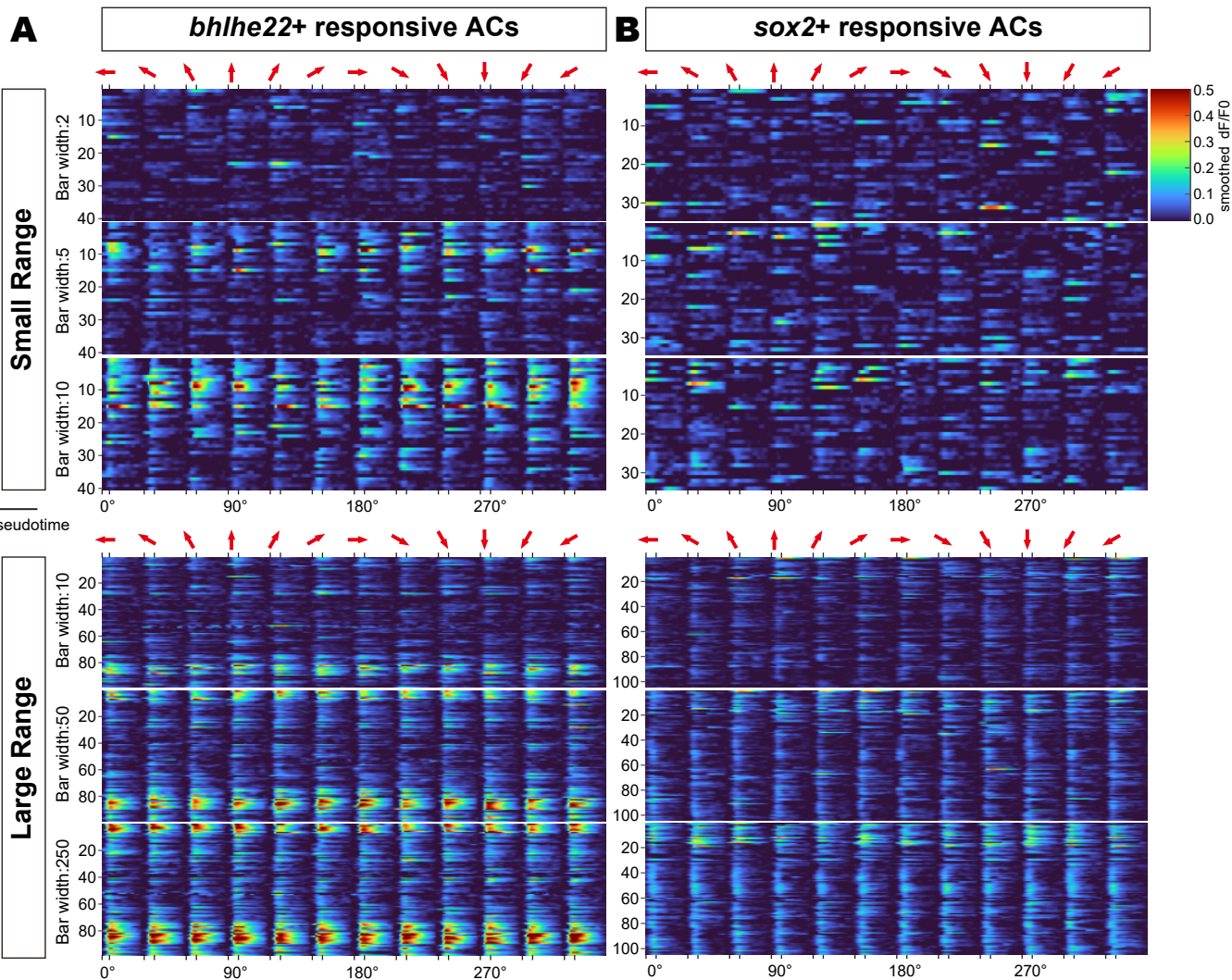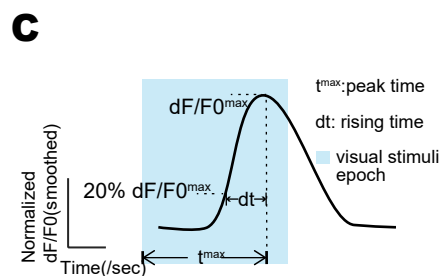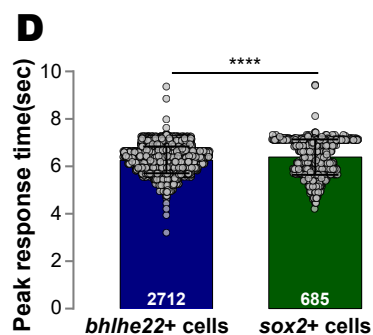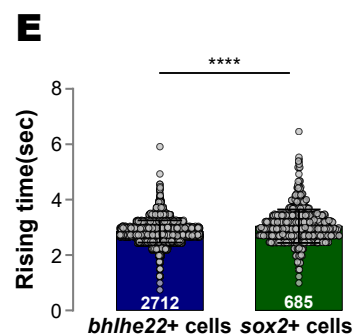

Supplement: S7 Fig — (A and B) Heatmap showing the response pattern of all responsive bhlhe22+(A) and sox2+(B) ACs in Fig 6E and 6F. (C) Schematic of a smoothed responsive trial for peak time and rising time. (D) Bar plot showing peak time of all smoothed responsive trials of 2 types of ACs as described in (C). (E) Bar plot showing rising time of all smoothed responsive trials of 2 types of ACs as described in (C). The data underlying this figure can be found in S3 Data. Data are presented as mean and SD. Mann–Whitney test. **** p < 0.0001. (PDF) [file pbio.3002506.s007.pdf]

**A**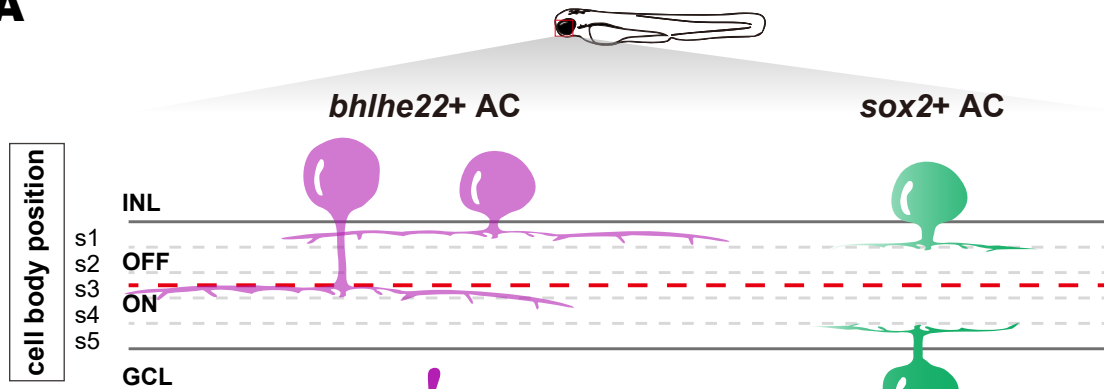**B**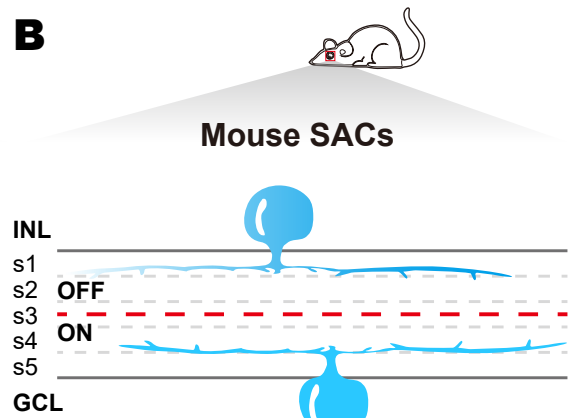**C**

dendritic distribution

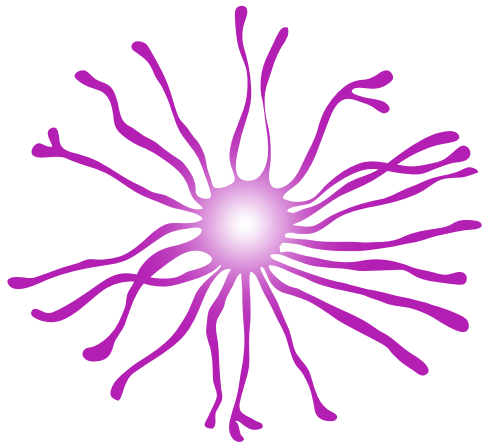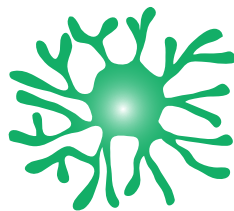**D**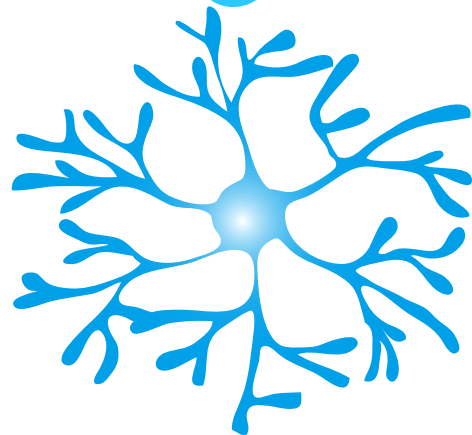

Supplement: S8 Fig — (A and B) Schematic showing cell-body positioning of zebrafish GABAergic/cholinergic ACs (A) and mouse SACs (B). (C and D) Schematic showing dendritic arborization in zebrafish GABAergic/cholinergic ACs (C) and mouse SACs (D). (PDF) [file pbio.3002506.s008.pdf]
