# Supplementary material for: Defining morphologically and genetically distinct GABAergic/cholinergic amacrine cell subtypes in the vertebrate retina
Source: PLoS Biol. 2024 Feb 16;22(2):e3002506. doi: 10.1371/journal.pbio.3002506 (PMC10914270; doi:10.1371/journal.pbio.3002506)

A

	bhlhe22	*bhlhe23*	*sox2*
At least one site disrupted	0.00%	1.52%	0.00%
At least two sites disrupted	6.82%	4.55%	0.00%
At least three sites disrupted	61.36%	12.12%	0.00%
Four sites disrupted	31.82%	72.73%	100.00%
Multiple sites mutated	50.00%	42.42%	89.71%
Site-spanning deletions	84.09%	60.61%	100.00%
Complex deletions/insertions	52.27%	65.15%	48.53%
> 50 bp insertions	11.36%	3.03%	1.47%
Total clone number	44	66	68

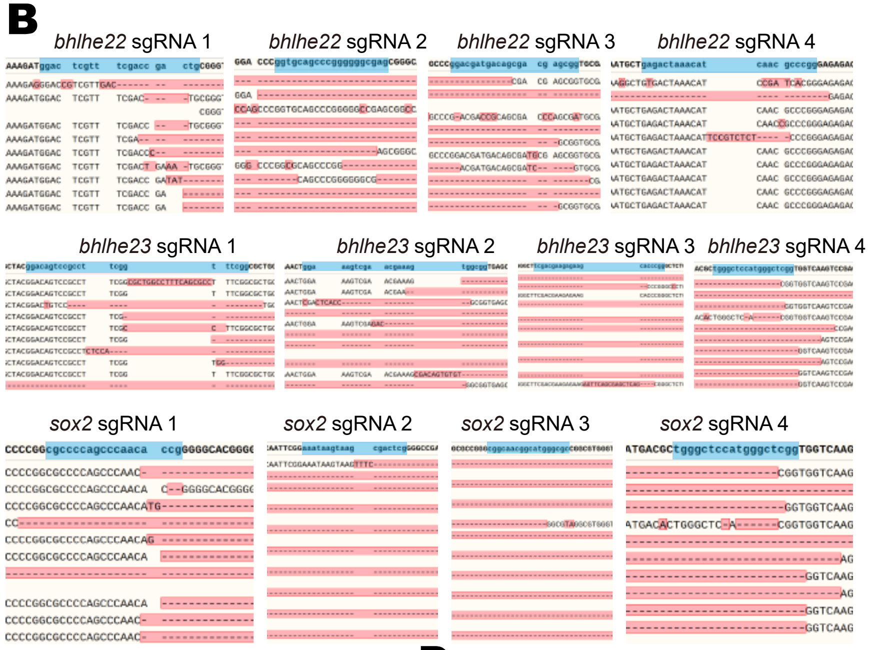

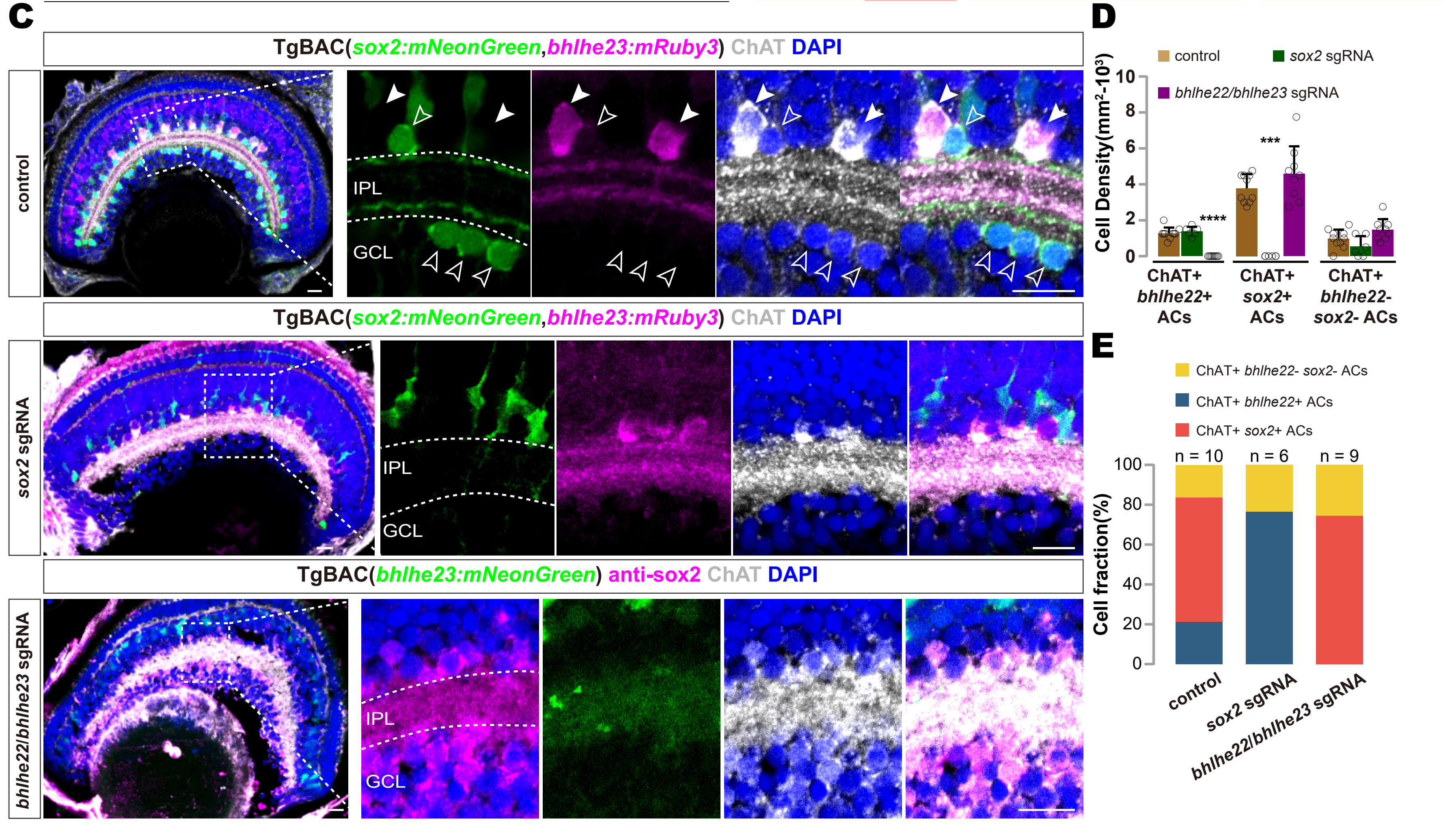

Supplement: S2 Fig — (A) Statistics of bhlhe22, bhlhe23, and sox2 disruption efficiency and gene disruption pattern through 4 CRISPR/Cas9 ribonucleoprotein complexes injection in founder embryos. (B) Examples of 10 alleles around 4 targeted sites of ORF of bhlhe22, bhlhe23, and sox2. First line is the WT sequence. Blue rectangle, sgRNA-targeted sites; red rectangle, mismatches. (C) Representative images showing ChAT immunostaining pattern of TgBAC(sox2: mNeonGreen,bhlhe23:mRuby3) of wild-type (up) and sox2 disrupted (middle) larval fish, and representative images showing SOX2 and ChAT immunostaining pattern of TgBAC(bhlhe23: mNeonGreen) after bhlhe22 and bhlhe23 codisrupted (bottom). Hollow white arrow head indicated ChAT+ sox2+ cells and solid white arrow head indicated ChAT+ bhlhe22+ cells. (D) Bar plot showing cell density of 3 types of cholinergic cells (ChAT+ cells) in (C). (E) Bar plot showing cholinergic cell composition of 3 groups in (C). The data underlying this figure can be found in S3 Data. Data are presented as mean ± SD, Mann–Whitney test. *** p <0.001, **** p <0.0001. Scale bars, 10 μm. AC, amacrine cell; ChAT, choline acetyltransferase; ORF, open reading frame; sgRNA, small guide RNA; WT, wild type. (PDF) [file pbio.3002506.s002.pdf]
