# Supplementary material for: Defining morphologically and genetically distinct GABAergic/cholinergic amacrine cell subtypes in the vertebrate retina
Source: PLoS Biol. 2024 Feb 16;22(2):e3002506. doi: 10.1371/journal.pbio.3002506 (PMC10914270; doi:10.1371/journal.pbio.3002506)
Supplement: S4 Fig — (A) Schematic design (up) of TgBAC(bhlhe23: gal4,uas:kaede) and the representative image (down). (B) Schematic design (up) of TgBAC(sox2: gal4ff,uas:kaede) and the representative images (down). (C) Representative images showing the ON and OFF subtype dendritic morphology of bhlhe22+ and sox2+ ACs. Images are captured from larval fish of 4 to 5 dpf. Scale bars, 10 μm. (PDF) [file pbio.3002506.s004.pdf]

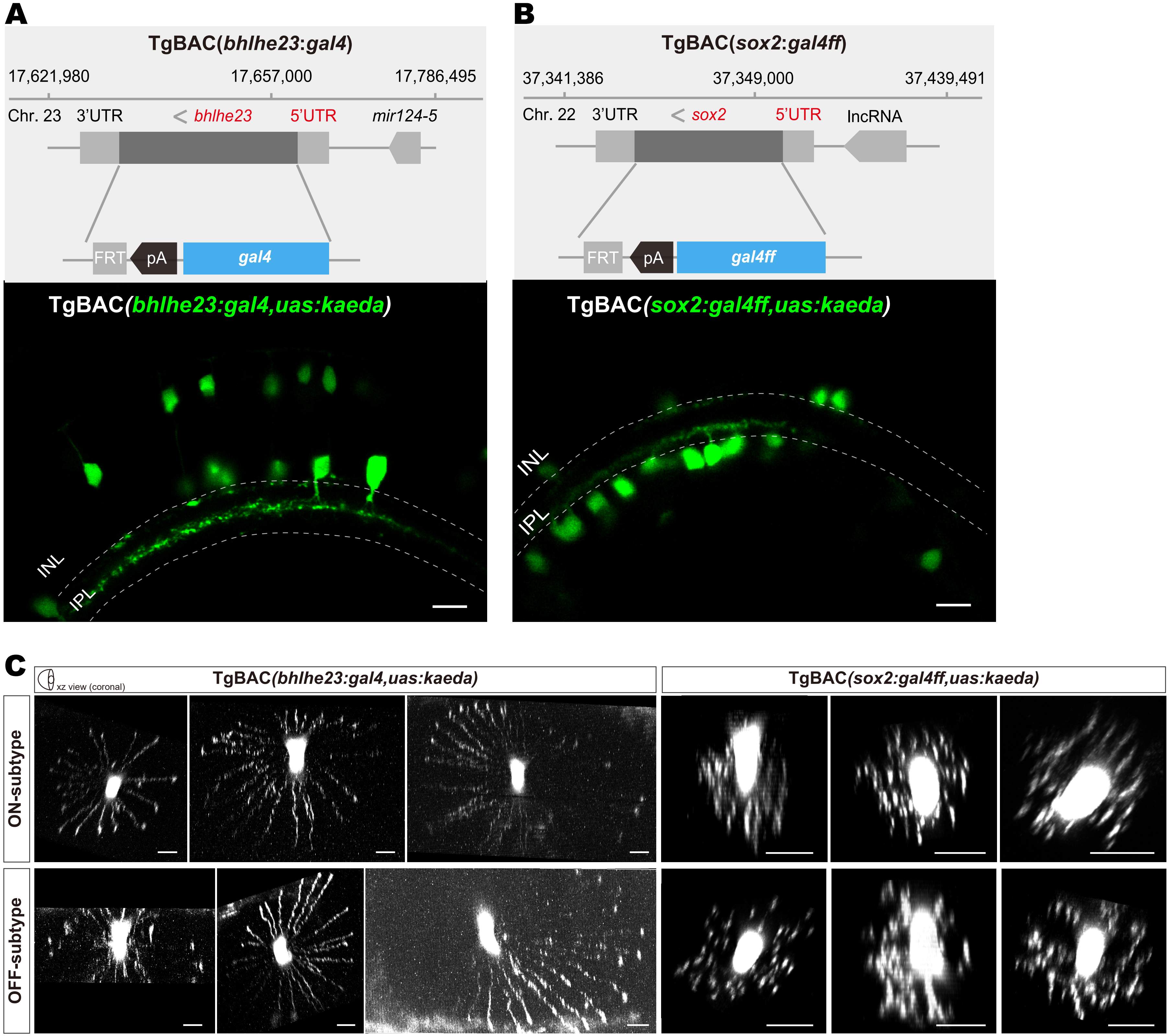
A
TgBAC(bhlhe23:gal4)
17,621,980
17,657,000
17,786,495
Chr. 23
3'UTR
< bhlhe23
5'UTR
mir124-5
FRT
pA
gal4
TgBAC(bhlhe23:gal4,uas:kaeda)
INL
IPL
B
TgBAC(sox2:gal4ff)
37,341,386
37,349,000
37,439,491
Chr. 22
3'UTR
< sox2
5'UTR
lncRNA
FRT
pA
gal4ff
TgBAC(sox2:gal4ff,uas:kaeda)
INL
IPL
C
xz view (coronal)
TgBAC(bhlhe23:gal4,uas:kaeda)
TgBAC(sox2:gal4ff,uas:kaeda)
ON-subtype
OFF-subtype
