# Supplementary material for: Defining morphologically and genetically distinct GABAergic/cholinergic amacrine cell subtypes in the vertebrate retina
Source: PLoS Biol. 2024 Feb 16;22(2):e3002506. doi: 10.1371/journal.pbio.3002506 (PMC10914270; doi:10.1371/journal.pbio.3002506)
Supplement: Attachment — Submitted filename: Replies to reviewers_Li et al.1017.docx [file pbio.3002506.s019.docx]

REVIEWS:
From Q1 to Q27, 27 questions
Reviewer #1, Juan Angueyra (note, Reviewer 1 has signed this review):
SUMMARY.
Li et al. describe two genetically and morphologically distinct amacrine-cell (or AC) types to have a role in direction selectivity in the zebrafish retina. Like the mouse starburst amacrine cell (or SAC), the sox2+ AC seems to be a symmetrical, narrow-field AC that is GABAergic and cholinergic. Interestingly, the authors identified a novel direction-selective AC that is also GABAergic and cholinergic and that shows direction-selectivity; this novel AC is characterized by expression of the transcription factor *bhlhe22*+. Both cell types can respond to moving bars of various sizes; the *bhlhe22*+ cells seem to prefer larger moving stimuli in comparison to the *sox2*+ cells. The authors show convincing differences between these two cell types by transcriptomics, genetics, morphology and function, providing a well-rounded starting point for future characterization of cholinergic cells and direction-selectivity in the zebrafish retina. The experiments are thorough, well-controlled
and analyzed. Collectively, the authors provide an insightful and significant piece to the expanding understanding of retinal-cell type diversity in vertebrates. The optokinetic and optomotor responses have become standard assays to evaluate visual function and perception across vertebrate animal models; understanding how direction selectivity arises in the retina is crucial to inform our understanding of the central processing of visual information.

SUGGESTIONS.
We consider that the results presented here very convincingly delineate the differences between these novel subtypes of cholinergic cells in the zebrafish retina. As the paper stands, the transcriptomic, genetic, morphologic data and the mutations in the respective transcription factors clearly support the main claims of this paper. These results are important for out field.

Most of our concerns lie in the characterization of the direction selectivity. We explain these below.

**Part 1: Selection of responsive cells**

**Q1:** **It is difficult to understand if the population data presented in Figure 4 derives from single ROIs, or if they represent averages across the 3 ROIs for a single cell. Methods mention "To evaluate the variance of preferential directions of various local dendritic regions from each cell，the standard deviation of tuned direction of cells with responsive ROIs more than 2 was analyzed" but these data are not presented or mentioned in results. Given the mammalian work on SACs, it would be presumed that individual dendrites may work independently and have disparate preferred directions, so that ROI data should not be pooled. Please make clarifications and consider changing labels from cell to ROI where appropriate**

Answer: Thanks for these important comments.

Previous studies have demonstrated that the direction-selective property of a neuron could be determined by direction-selective responses of its local dendrites [1-3], despite that individual dendrites were presumed to work independently and have disparate preferred directions[3-5]. In the revised manuscript, we have updated the method of ROI selection. We analyzed all ROIs on individual dendrites of each responsive neuron and calculated the DSI of single ROIs. The DS property of two AC types was estimated by the DSI distribution of single ROIs derived from either AC type (*bhlhe22*+ ROIs and *sox2*+ ROIs). We have made further clarifications in the revised Materials and Methods (Lines 146-149 in supporting information).

**Part 2: Selection of ROIs**
**Q2: What was the random selection process for ROIs?**

Answer: Thanks for the important question.

In this new revision manuscript, we have updated an unsupervised procedure to select all ROIs on local dendrites. Details have been clarified in the revised Materials and Methods in Lines 146-149 in supporting information.

**Q3: Is 0° standardized between larvae? is it rightward motion? please explain**

Answer: Thanks for the questions.

Yes, 0° is standardized across all samples, and it is leftward motion in our experiments. We have updated the information in the figures (fig.5, fig. S5 and S7) in the revised manuscript.
 **Q4: Text describes that the response in sox2+ cells is smaller than for bhlhe22+ cells but example panels B and D in Figure 4 show the opposite**

Answer: Thanks for raising this question.

We have revisited our data. In the revised manuscript, we applied a more rigorous procedure to calculate ROI responses, and we also removed the outliers from the dataset using Graphpad (Details in Materials and Methods Lines 145-146 in supporting information). The new result showed that the amplitude of responsive *bhlhe22*+ ROIs was statistically smaller than responsive *sox2*+ ROIs (Fig. S5C), despite that the response amplitude of the soma was statistically larger in *bhlhe22*+ cells than in *sox2*+ cells (Fig. 6E and 6F).

**Part 3: Possible direct stimulation of cells**

**Q5: How was the size of the bars determined**

Answer: Thanks for raising this important question.

An earlier study has shown that the larval fish could exhibit optokinetic reflex responses to the shift grating with the spatial frequency at 0.033 (equivalent to approximately 50-pixels width bars; visual angle: 50° given visual distance of 0.36 cm) [6]. In our OKR assay, we also confirmed that the larvae fish exhibited OKR responses to shift grating with 50-pixel-width bars (visual angle: 50° given the visual distance of 0.36 cm). Thus, I decided to use 50-pixels-width bars in further experiments.

**Q6: How does the size of the bar match the receptive field, or the dendritic arbor, of both cell types?**

Answer: Thanks for raising the important comment regarding the receptive field.

First, as described above in Q5, we determined the optimal bar size (50 pixels; 3.25 mm; equivalent to the visual angle of 50 degrees given the visual distance of 0.36 cm) based on the previous study and our result of the OKR assay, not the receptive field or the dendritic arbor.

To address the relationship between the bar size and the receptive field (or the dendritic arbor), we made an estimation in the revised manuscript. Take 50-pixel bar size as an example. Given: 1) the bar size of 3.25 mm; 2) the visual distance of 3.6 mm; 3) the distance between the lens and the retina is 100 microns, the size of the 50-pixel bar projected on the retina is estimated to be about 90 microns. Considering the dendritic arbor sizes of bhlhe22+ ACs and sox2+ ACs are ~90 microns and ~30 microns, respectively, for a rough estimate, 50-pixels bars can cover the dendritic arbor of one bhlhe22+ AC and three sox2+ ACs, respectively.

**About Q7: In our experience, while recording calcium-responses in the retina in a PTU-treated fish, it is possible (and likely) that stimulation of photoreceptor occurs directly with light that traverses larval tissues and enters the back of the eye (which has been made transparent) and is absorbed by outer segments. Most of the optic tectum work is perhaps protected from this issue because they tend to keep eye pigmentation. We have recorded robust retinal responses in larvae positioned on their side and with stimulation through the condenser, which precludes light stimulating the retina by entering through the lens.
About Q7: Given the size of the dendritic fields of these cells (less than a 100 microns), and the speed of the bars (16300 microns per second) and screen refresh rate (60 Hz?), the bar edge moves ~270 microns between frames, potentially covering all cones that provide input to these cells in a single frame (if they were stimulated directly and not by light entering through the lens)
Q7: Can the authors make any claims about the location of each cell’s receptive field with the available data? Can these be estimated?**

**Q8: Alternatively, can the authors test if direct stimulation of photoreceptors is an issue by flashing small spots underneath the larvae? Or, can the stimulus be blanked underneath the larvae while direction selective responses are being measured?**

Answers: Thanks for raising these insightful comments regarding the stimulation.

We appreciate the idea that the reviewer shared here (“We have recorded robust retinal responses in larvae positioned on their side and with stimulation through the condenser”). We also agree with the review’s concern. In our experiment design, we cannot completely rule out the possibility of direct stimulation of photoreceptors with light that traverses larval tissues in PTU-treated larval.

However, several lines of our result evidence suggest that even if such direction photoreceptor stimulation present, it is unlikely to significantly influence the direction selectivity of two AC types: 1. Responses of two AC types were highly selective (12% of 4176 *bhlhe22*+ ROIs showed direction-selective responses, 24.2% of 2338 *sox2*+ ROIs showed direction-selective responses), suggesting the high specificity of photoreceptor activation by light instead of the overall cone stimulation. 2. In our experimental setup, the stimulation light is given from the ventral side of the larval, whereas the imaging is mainly carried out at the dorsal side of the retina (Fig. 5A), which reduced the influence of direct photoreceptor stimulation by the light, although it could not rule out this possibility. 3. Also, in a previous study [7], the light stimulation was delivered directly at the lateral side of the eye to elicit direction-selective response in the PTU-treated larval fish.

Together, although we could not rule out the presence of direct stimulation of photoreceptors by the light transversed through the tissue, our experimental setup and the high selectivity of direction-selective responses derived from two AC types limited the contribution of the light transversed through the tissues to our conclusions.

**Part 4: Size selectivity**
**Q9:** **For size selectivity and Figure 6, the analysis of DSI seem to switch from individual ROIs to cell bodies. Is this correct? Can the authors explain the rationale behind this decision?**

Answer: Thanks for the comment.

Yes, the analysis of DSI in Figure 6 was based on responses of cell bodies. The previous study has demonstrated starburst cells could in principle integrate inputs across fairly substantial fractions of their total dendritic arbors [8], and the previous electrophysiological study also showed that cell bodies of amacrine cells could showed directional-selective responses [9, 10]. Also, individual dendrites showed the different preferential directions, which raises the challenging to DSI estimation of a neuron. Thus, the estimation of DSI using soma responses rather than dendritic local responses became much more straightforward.

More importantly, in Figure 6, we focused on the responses to different bar sizes, which mainly represents responses integrated from the entire receptive fields. Therefore, we recorded calcium responses of the soma instead of local dendrites. In addition, the analysis of DSI based on cell bodies’ responses also improved the experimental throughput.

**Q10:** **In panels I and K, the calculated DSI are above 0.20 for the example cells. For what bar width are these example responses?**

Answer: Thanks for this important question.

We have updated the original panel I and K as Fig S7C and S7D in the revised manuscript. All the response of direction-selective cells in all size have been shown (S7E-S7G Fig).

**Q11:** **The average DSI across all bar widths is well below 0.20 (Panels M and N). Are the example traces from far outliers in the dataset?**

Answer: Thanks for raising this important question.

In the previous version, the average DSI was calculated for all responsive cells instead of direction-selective cells, which led to the average DSI well below 0.2. We have updated the part of analysis in the revised manuscript. The new analysis showed that the average DSI of direction-selective *sox2*+ ACs and *bhlhe22*+ ACs was 0.74±0.18 and 0.65±012, respectively(Fig.S7G). Also, we showed all response traces of direction-selective cells in the revised Fig. S7C and S7D.

**Q12:** **If DSI threshold in Figure 5 was 0.1, should the interpretation of these data be that there is little to no direction-selective responses to these bars as measured in this figure?**
Answer: Thanks for raising this important concern.

In the revised manuscript, we have applied more rigorous criterion for DSI to determine the directional selectivity. In previous studies, the threshold of DS tuning was set in the range of 0.3 to 0.5 [11-16] . In this revised version, we set the DS tuning threshold to 0.5. The vector sum was calculated as the summation of these normalized response vectors [17]. The direction selectivity index (DSI) was calculated as follow:

$$DSI = \frac{Pref - Null}{Pref+Null}$$

where Pref is the response in the stimulus direction closest to the preferred direction and null is the response in the opposite stimulus direction. The preferred direction was obtained from the direction of the vector sum[17].

We have updated related results in manuscript in Lines 183-318 and method part in Lines 156-167 in materials and methods in supporting information.

**Part 5: Statistical analysis**
**Q13: Mann-Whitney tests are appropriate for single pair-wise comparisons but are likely to give false positives when repeatedly used for multiple pairwise in groups larger than 2. - Consider using a Kruskal Wallis test and a subsequent post-hoc test where appropriate.**
Answer: Thanks for these great suggestions.

In this revised version, we have applied Friedman test and subsequent Dunn’s multiple comparisons test to pairing wise (Fig.6E-6H), as well as Kruskal Wallis test and subsequent Dunn’s multiple comparisons test using GraphPad Prism (8.4.3) to multiple unpair wise in groups larger than 2 (Fig.S7E and S7F).

**Q14: Line 88: " At 72 hpf, retina development is thought to be mostly completed in zebrafish [38]" This statement should be modified. At 72 dpf, most retinal cells are still quite immature; a prime example are photoreceptors which have short outer segments, poor light responses correlating to low expression of phototransduction genes and with a mosaic that is very disorganized.**
Answer: Thanks for the important suggestion.

We have corrected it in the revised manuscript in Lines 90-91.

**Q15:** **Line 178 - 186: It is not made clear for readers that the experiments for "The generation of two AC types requires distinct sets of TFs" section derive from F0/G0 mutants, which are genetic mosaics, and not germ line mutants.**
Answer: Thanks for the important comment. We have updated it in the revised manuscript in Lines 130-131.

**Q16:** **The variability is dendritic field size of bhlhe22+ cells is quite large. The smaller cells in this "wide-field" class rival the size of the sox2+ "narrow-field" cells. These all correspond to 5 dpf larvae, correct? Is there a systematic difference between the ON and the OFF subtypes? Is this same range seen in cells that responses were recorded from? Do responses match dendritic size characteristics at all?**
Answer: Thanks for the comment.

1) Yes, all of the field size corresponds to 5 dpf larvae.

2) For *sox2*+ cells, the diameter of OFF-laminae subtype sox2+ cells (31.63±4.67 μm) were slightly larger than ON-laminae subtype (27.95±4.13 μm, p = 0.042), whereas the area of the two subtypes did not show significant difference.

3) For *bhlhe22*+ cells, the diameter and area of OFF-laminae subtype did not show significant difference with ON-laminae subtype.

**Q17:** **The authors suggest the evidence illustrates sox2+ cells in zebrafish to be the evolutionary counterpart to the mouse SAC cells. Authors may consider assessing the coverage factor and tiling of the sox2+ cells in the zebrafish. Are the sox2+ cells as densely tiled as the SACs in mice? What about the bhlhe22+ cells?**

Answer: Thanks for these important suggestions. In our analysis, we have showed that *sox2*+ ACs more-likely resembled mouse SACs in terms of fate-determinators and lamination organization compared to *bhlhe22*+ ACs (Fig.3B,4A,4B).

It is a brilliant idea to find more similarity in coverage factor and tiling of zebrafish and mouse *sox2*+ ACs as the reviewer proposed. Unfortunately, we still do not manage to do this due to the following reasons: 1. we found the dendritic arbor size of both AC types (*sox2*+ and *bhlhe22*+) was continuously growing till the adult stage (data not shown). It is likely due to the fish-specific life-long growing of the whole-body tissues. This also suggests the coverage factor and tiling of two AC types may change with age in zebrafish, which could be different from the mouse. 2. More importantly, according to the definition of the coverage factor used in previous mouse studies [18, 19], the factor of a dsRGC is determined by the number of SACs that the dsRGC contacts. However, so far it is technically difficult to mark individual dsRGCs and either AC type simultaneously in the zebrafish retina.

**Q18: Figure 2, panel F: there is not enough information to decode this panel in results or legend. The description for panel F is duplicated and not very informative. Why are there 2 bars? what is 100% for each one? why do they share an n?**

Answer: Thanks for the comments.

It was updated in the revision manuscript Fig 2F. n is the number of fish, which were offsprings of TgBAC(*bhlhe22:mNeonGreen*) outcrossed with TgBAC(*bhlhe23*:*mRuby3*). This is why they share the same number.

**Q19: Figure S1, panel H: what does "dAC" stand for?**

Answer: Thanks for the comment.

"dAC" stands for displaced amacrine cell. We have updated this in the revised manuscript Line 764.

**Q20: Figure 4, panel E: "Placed" is an awkward term for this. Perhaps: "Cell body in INL" vs. "Cell body in GCL (displaced)"?**

Answer: Thanks for the comment.

We have updated it in the revised manuscript in Lines 708-713.

**Q21: Figure 4, panel G: the data suggests that there is a loss of cells on average and not just a lack of displaced cells. Are the differences in the sum of ON and OFF sox2+ cells significant?**

Answer: Thanks for the comment.

The differences in the sum of ON and OFF *sox2*+ cells are not significant after the loss of *fezf1* (Fig. 4H).

**Part 6: In Methods**
**Q22: Line 59: How long were samples fixed for?**

Answer: Thanks for the comment.

Samples were fixed at 4°C for about 8 hrs. We have updated it in the revised Materials and Methods Line 60 in supporting information.

**Q23: Line 66 - 68: provide details about antibodies including species, catalog and lot number.**

Answer: Thanks for the comment.

We have updated it in the revised Materials and Methods Line 67-77 in supporting information.

**Q24: Missing details for OKR: how far was the cylinder from the larvae? how fast did the grating move? was it reversed? for how long? can you provide details about the screen (make, spectral compositions, etc.)?**

Answer: Thanks for the comment. In the revised manuscript, we have included all these information in the revised Materials and Methods (Lines 113-116) in supporting information.

**Q25: Missing details for Visual stimulation under 2P: How far was the larvae from the screen surface? how many degrees of visual angle are covered by the screen?**

Answer: Thanks for the comment.

Vertical distance from larvae lens to the screen was 0.5 mm (visual angle:178°×179°). Details was updated in revised Materials and Methods in Line 140-141 in supporting information.

In general, the introduction and methods could use style editing and grammar corrections that would help with clarity. A none comprehensive list is below.

**Part 7: MINOR EDITS**

Figure 2, panel K: remove yellow dotted outline in Gad65/67 panel

Figure 4 and Figure S4, panel A/B: kaede is mispelled
Line 37 - change to "… (SACs), which are also cholinergic [3-8]." Line 42 - 44 - change to "in the mouse retina, expression of Sox2 and Fezf1 were required for appropriate cell body positioning of ON SACs [12,13]."

Line 44 - add coma "showed that OFF, but not the ON subtype,…"

Line 45- 46 - Run on sentence. Modify to "…from non-SACs. These inputs, involved in centrifugal direction selectivity, suggest different…"

Line 49 - Change "were" to "are" Line 58 - Unclear wording. Change to "…[22-24]; which are a diverse cell type [25,26]."

Line 60 - Add semicolon. "…neurite arborization; including: narrow-field…"

Line 62 - delete "either" Line 62 - Run on sentence. Change to "…[29]; however, there are a few amacrine cells that…"

Line 65 - Organization. Structure to " …arbors with some overlapping and are born earlier…"

Line 66 - "Cholinergic ACs are a subpopulation of GABAergic ACs"

Line 70 - 71 - Modify wording to "Additionally, we performed…"
Line 74 - change "further" to "furthermore" Line 80 - wording unclear. Change "showed higher responses" to "responded more robustly to…"
Line 81 - Change "the broader size range…" to "a broader size range"
Line 89 to 91 - Change to: "From the 19 retinal clusters we identified 5 AC clusters based on co-expression of elval3 and tfap2a; these 5 AC clusters could be readily differentiated by the expression of marker genes (...)"
Line 95 - Noun is singular. Change "were" to "was"
Line 96 - define "MC" here.
Line 98 - Use ChAT. Already defined acronym in line 52 Line 100 - delete "were" in the area: "type were resided in both…"
Line 125 - Consider changing "mark" to "identify"
Line 126 - delete "either"
Line 126 - mNeonGreen is misspelled Line 128 - rephrase for clarity. "…specifically marked a subset of ChAT+ and Gad65/67+ ACs located in the INL…"
Line 129 - add "the" - "together with the in situ results…"
Line 130 - rephrase for clarity. "…specifically marked the bhlhe22+…"
Line 131 - rephrase for clarity.
Line 132 - add semicolon. "…bipolar cells(Fig 2E and 2F); which is consistent…"
Line 133 - rephrase for clarity. "specifically marked a ChAT+ and Gad65/67+ subset of ACs located in the INL…; which is consistent with…"
Line 135 - reword for clarity. "…were co-labeled with a SOX2 antibody…"
Line 137 - Change "besides" to "additionally"
Line 139 - Reword for clarity and add "the" before the word "two". "Furthermore, crossing TgBAC… show that the two AC types…"
Line 211- Unclear sentence. Reword for clarity.
Line 212 - Change 'it' to 'this'
Line 212 - Change wording. "…we examined the influence of disrupting bhlhe22/bhlhe23 or sox2…"
Line 217 - Reword for clarity. "we could not…that SoFal lacked the sensitivity to detect changes within retinal subtypes."
Line 223 - add "lines" - "using newly-generated lines…"
Line 225 - add "the" - "…, the dendrites…"
Line 226 - reword for simplification. "… starburst-like pattern reminiscent of mouse SACs [16]." Line 316 - use 'OKR' abbreviation already defined in line 75.
Line 316 - change "is the behavior" to "is a behavior" Line 390 - change 'it' to 'this' Line 459 - change 'the' to 'a' Line 468 - Reword for clarity. Change 'higher' to 'more robust'
Line 473 - Simplify wording. "which express sox2. [43]."
Line 474 - add colon. - "… including: cat…"
Line 475 - Simplify for clarity. Start sentence at "Although cholinergic ACs are observed in many non-mammalian species, such as: chick…"
Line 479 - Consider using different wording for 'placed' and 'displaced' cells or clearly defining them in this context.
Line 480 - Consider rewording for clarity. "led to inappropriate soma placement in the INL" ?
Line 499 - delete 'to' - "presynaptic bipolar cells (BPs)…"
Line 503 - add 's' to AC Line 505 - delete 'and' - "…, thereby resulting in…"
Line 506 - what does 'more-fined" mean here? Consider rewording.
Line 507 - add 's' to population
Line 508 - Reword for clarity and simplification. "…subtypes also lack specific ON or OFF responses."
Line 509 - Run-on sentence. End at "response." - "This requires future efforts…"
Line 509 - change 'in' to 'of' - "difficulty of genetically marking…"
Line 514 - change 'on' to 'of' - "the advantage of…"
Line 517 - delete 'the' - "… generated larger responses…"
Line 517 - delete 'of' - "…same size objects…"
Line 518 - add 'the' - "… the larger dendrites…"
Line 518 - change 'titling' to 'tiling'
Line 520 - add 's' to type Line 521 - delete 'either'
Line 521 - Change wording for clarity. Change 'more' to 'additional' or 'more numerous'
Line 522 - change 'side' to 'size'
Line 522 - reword for clarity. " …could enhance the response of the bhlhe22+ type as a function of bar size."
Line 526 - Change 'may better receive' to 'may receive more'
Line 526 - delete "dendrites, which could result" change to 'dendrites resulting in an…"
Answer: Thanks for all these constructive suggestions. We greatly appreciate the reviewer’s effort on these edits. We have updated all of them in the revised manuscript.

From Q26 to Q36, 11 questions
Reviewer #2, Takeshi Yoshimatsu (note, Reviewer 2 has signed this review): This manuscript by Li Y, et al. reports the discovery of two types of direction-selective (DS) amacrine cells in larval zebrafish. The genetic approaches to identify DS amacrine cells are very thorough and the amount of work done is impressive. In particular, the finding that one of the DS types is similar to the mammalian DS amacrine cells (aka starburst amacrine cells) in terms of the molecular marker and the developmental origin (fexf2 dependent cell locarization) is interesting. The authors further characterize the morphology and the properties of direction selectivity in these DS amacriine cells and found that one type has smaller dendritic field size and responds robustly to smaller moving objects, whereas the other type has larger dendritic field size, larger responses, and tuned to larger moving bars. Finally, the authors tested the roles of these DS amacrine cells in visual behaviors using an
optokinetic response assay. This revealed that that both types are required for the normal frequency of saccade eye movements, but not for the normal saccade amplitude. Taken together, this manuscript reports the conserved and a novel type of DS amacrine cells in the vertebrate retina. These findings are of interest to broad audience including retinal physiology and visual ecology. However, I find that the Methods section in the supporting information needs much improvement and some clarification is required in the results due to the lack of some information in the methods.
**Q26: Did the authors verify the sequences of the riboprobe? According to the methods section, the DNA template for the probe synthesis was prepared by PCR reaction from cDNA. This means that the DNA template sequence was not confirmed.**

Answer: Thanks for the comment.

Yes, we verified the sequences of PCR products of riboprobes. The data was included in the Fig S1F and revised Materials and Methods Line 85 in supporting information.

**Q27: The visual stimuli parameters are unclear. For object size preferential test, the bar widths are described as (bar width: 2,5, and 10 pixels, 0.07 cm/pixel) for the small range. However, in Fig. 6 E, there are three bar widths used. For the big range, what was the size of the pixel? Finally, the size on the screen is not very informative. The authors should provide the size in the visual angle.**

Answer: Thanks for the comment.

For object size preferential test, moving bar size in small range (bar width: 2,5, and 10 pixels, visual angle: 1°/pixel at visual distance of 0.36 cm) and big range (bar width: 10, 50, and 250 pixels, visual angle: 1°/pixel at visual distance of 0.36 cm) in full field of screen were applied. We have updated this in revised Materials and Methods in Line 116-122 in supporting information.

**Q28: Are the 10 pixel bar sizes in the small range and the large range the same? If so, why are the response amplitudes, responsive cell fractions, and DSI different in Fig. 6E-H, M,N?**

Answer: Thanks for raising this issue.

Yes, the visual stimuli parameter of bar size 10-pixels in the small range and the large range is the same. We have updated Fig.6 and Fig.S6 in revised figures. We also noticed this difference of result of 10-pixel bars between the small range and the large range. One possibility is the influence of other bar sizes that were grouped with 10-pixel bars in either the small or large ranges on the response properties of 10-pixel bars. However, we do not understand the exact mechanism underlying.

**Q29:In the ‘in vivo two-photon calcium imaging’ section, the resolution is written as ‘60.42×57.88 μm’ (line 121). This resolution seems too big to resolve cells…**

Answer: Thanks for the comment.

The resolution is 0.636 × 0.636 μm/pixel (image size: 96×92 pixels). We apologized for this mistake. We have clarified this in revised Materials and Methods in Line 132 in supporting information.

**Q30: The authors should clarify the location of the cells recorded in in vivo two-photon calcium imaging. Because the visual stimuli were projected beneath the fish, this information is important to know the visual angle for a given bar width. For example, if the cell was located in the dorsal region of the eye, they see the bottom of the dish where the bars with the same width would appear wider in the visual angle compared to the bars further away which is seen by the cells located close to the center of the eye.**

Answer: Thanks for the comment.

In our imaging setup, all recoded cells were located at the dorsal side of the retina.

**Q31: This sentence is unclear ‘Time-series images process of calcium responses was performed described (132)’**

Answer: Thanks for the comment.

We have updated it to “The image processing of calcium responses was conducted as previously described [7].” in the revised Materials and Methods in Line 144 in supporting information.

**Q32: In the ‘Functionl analysis’ section, the authors used some criteria to identify “visual response”. However, the rationale to remove ‘3),trials with peak amplitude exceed 4×SD of 140 dF/F0 of all four trials’ as outliers are unclear.**

Answer: Thanks for the comment.

The previous study processed the outliers by Antinucci [7]. Since this study was done in the zebrafish retina, we decided to employ the same procedure to remove the outliners. We have clarified this issue in the revised Materials and Methods in Line 154-156 in supporting information.

**Q33: Line 141. ‘Peak amplitude of averaged value of all trials during visual stimuli was the raw response (dF/F0).’ This does not make sense. If this is true, there is one raw response per ROI.**

Answer: Sorry for the confusion.

We have clarified this issue in the revised Materials and Methods in Line 156-158 in supporting information. In fact, there is one raw response per ROI. Average value of trials after removing outliers was calculated, and it was the final raw response of each ROI. Peak amplitude during visual stimuli was set as the representative response (dF/F0) of the ROI.

**Q34: Line 144. How the DSI is computed is unclear. Where ‘r’ is computed?**

Answer: Thanks for the comment.

In the revised manuscript, we have updated the analysis of DSI as described previously[17]. The response ‘r’ was normalized to the summation of these normalized response vectors $\sum{(\frac{dF}{F0})}_{i}$. Where i, 1 to 12, corresponding to 12 directions spanning 0° to 330° at 30° interval. We have clarified the details in the revised Materials and Methods in Line 158-160 in supporting information.

**Q35: What is the reason to evaluate the variance of preferential directions? (Line 146)**

Answer: Thanks for the comment.

We have updated the analysis of DSI part in the revised manuscript. In this new version, we have analyzed all qualified ROIs on the local dendrites as described in Materials and Methods. Thus, it is no necessary to evaluate the variance of preferential directions, and we have removed the analysis of variance of preferential directions from the revised version. We have clarified the details in the revised Materials and Methods in Line 146-151 in supporting information.

**Q36: Line 148, the sentence ‘For calcium response… analysis.’ Is a duplicate of the sentence in line 134.**

Answer: Thanks for the comment.

We apologized for this mistake and have removed this duplication in the revised Materials and Methods Line 164 in supporting information.

From Q37 to Q48, 12 questions in total
Reviewer #3: This paper describes for the first time the presence of two amacrine cells (AC) types in the zebra fish retina that have molecular properties similar to cholinergic/GABAergic cells seen in mammalian retinas. Evidence points to a role of these cells in generating direction-selective responses within the zebra fish visual system. The circuitry generating direction-selectivity within retinal ganglion cells has been widely studied, mostly in mammalian species, as an example of complex neural processing. The paper presents some interesting new results and lays the groundwork for a comparative analysis of DS circuits in fish versus mammalian systems, which could be of general interest to neuroscientists.

The molecular characterization of the 2 novel AC types was convincing and well supported by the data. Calcium imaging of terminal dendrites demonstrated directional responses in these ACs, but control data showing the magnitude of direction-selectivity indices (DSI's) recorded from non-directional cells under the same conditions is missing.  Such control data seems essential to set the criterion DSI level for determining whether a cell is considered direction-selective or not. As it stands, the authors simply use a cut-off for the DS index of 0.1, which seems rather low. In any case, in the absence of control data, it would be helpful to know the rationale for selecting this 0.1 criterion level.

A focus of the paper is the finding that the two AC types have rather different dendritic field sizes. This observation prompted an analysis of the size-selectivity of the two AC types. Functional differences were observed between the two AC types, however, this section was not well motivated.  If the ACs are providing directional inhibition that generates directional responses in ganglion cells, it is not obvious why the cells should be tuned to the width of the stimulus bar. It would be helpful if the authors provided a stronger rationale or a proposed circuit model that could help the reader interpret the results and understand the implications of this size-sensitivity.

A key finding, linking the activity of the two ACs to the generation of directional responses in the fish visual system is supported by the OKR experiments shown in Fig. 5J-M. These results demonstrate a rather modest but significant effect of cell ablation on the saccade frequency during OKR. I do not agree that the results support a "major" contribution, particularly regarding the sox2+ cells. In any case, the authors don't explain why one would expect a change in the frequency of saccades when directional signaling is compromised. Perhaps a more informative metric would be to measure the gain of the OKR image tracking between saccades? See Fig 1 in Yonehara et al (2018) for an example in the mouse (https://doi.org/10.1016/j.neuron.2015.11.032).

Overall, there are some very interesting new observations, but in my view the paper could be greatly improved by a substantive revision.

**Q37: Calcium imaging of terminal dendrites demonstrated directional responses in these ACs, but control data showing the magnitude of direction-selectivity indices (DSI's) recorded from non-directional cells under the same conditions is missing.**

Answer: Thanks for the comment.

The DSI was calculated in an updated method in the revised manuscript. Although the threshold of DS tuning was variously set in previous studies, from 0.3 to 0.5 [14-19], we set the DS tuning threshold to 0.5 in this updated revision. DS representative traces of visual stimuli of bar width 50 pixel larger than DSI 0.5 has been updated in the revision manuscript. We also provided DSI value of non-directional cells in the revised manuscript. DSI value of non-directional *bhlhe22*+ cells and *sox2*+ cells are 0.20 ± 0.15 and 0.22 ± 0.17 respectively (S5G Fig, Lines 200-202). Data was in mean ± S.D.

**Q38: If the ACs are providing directional inhibition that generates directional responses in ganglion cells, it is not obvious why the cells should be tuned to the width of the stimulus bar.**

Answer: Thanks for this interesting question. We are curious about the implication of *bhlhe22*+ ACs with dendritic arbors significantly larger than *sox2*+ SACs in the zebrafish retina. Since the previous study showed that larger dendritic arbors gave rise to larger receptive fields[20], *bhlhe22*+ ACs are likely to have receptive fields larger than *sox2*+ ACs. As a result, two AC types exhibit response tuning to different objective size ranges.

Also, we are quite curious about to what extent *bhlhe22*+ ACs are conversed in the retina of other vertebrate species. Also, it is possible that the presence of *bhlhe22*+ ACs have been evolved to adapt to aquatic environments specifically for fish species.

**Q39**: **Authors don't explain why one would expect a change in the frequency of saccades when directional signaling is compromised.**

Answer: Thanks for raising this important question. Direction-selective circuits, consisting of direction-selective starburst amacrine cells and direction-selective retinal ganglion cells, underlie OKR anatomical pathways[21, 22]. The connection from DS circuits in the retina to the brainstem is required for the eye saccade movements[23]. Also, retinotectal DS inputs are required for the habituation of the OKR [24]. Thus, an impaired DS circuit in the retina could change the saccade's frequency or amplitude.

**Further comments and suggestions for revision
Q40: Line 226: the resolution of the cell fills is insufficient to suggest that the sox2+ cells had a starburst-like morphology reminiscent of putative homologous cells in mouse. Individual dendritic branches are not clearly resolved, making such a judgement questionable.**
Answer: Thanks for the comment.

It has been updated in revision manuscript (Fig.4A).

**Q41: Line 227, Fig. 4B: The authors note that the dendrites of the bhlhe22+ ACs are 10 times larger than the sox2+ cells.  The area of the dendritic arbor is 10-fold larger, but the dendrites are only ~2.5-fold longer. It is more conventional and useful to quote linear dimensions rather than area. This is particularly relevant to cells potentially involved in mediating direction-selectivity, which involves spatio-temporal interactions across the retina that depend on distance rather than area.**

Answer: Thanks for the comment.

We have included the information of linear dimensions in the revised manuscript (Fig.4C).

**Q42:** **Fig. 4E: There are no "displaced" bhlhe22+ ACs in this panel. According to the text "placed" somas are in the GCL, yet in panel C the bhlhe22+ cells are all "displaced" to the INL?**
Answer: Thanks for the comment. We have further clarified this in the revised manuscript (Fig 4C-F). According to the dendritic stratification laminae in the IPL, the *bhlhe22*+ ACs and *sox2*+ ACs were classified to ON-laminae and OFF-laminae subtypes.

**Q43: Fig. 4F: the finding is that the disruption of fezf1 causes the displacement of the On-type sox2+ AC somas to the INL. I assume that the On- and Off-type sox2+ ACs were distinguished according to the placement of the somas, with On-type in the GCL and Offs in the INL? In control, the sox2+ dendritic band in Sublamina-4 (S4) is stronger than the band in S1 (Fig. 4F), consistent with the 3-fold higher cell count for On-sox2+ ACs shown in Fig. 4G. However, although the S4 band has largely disappeared in the fezf1 retinas, as expected, one would expect to see a stronger band in S1, but this is not observed. Why are the dendrites of the displaced S4-ACs and S1-ACs missing from this image? Is this a representative result, and if so, is it possible that there are concomitant changes in dendritic arborization in the fezf1 retinas?**
Answer: Thanks for the comment. Higher-resolution images of the control and fezf1-disrupted groups were included in the revised manuscript (Fig.4G-4H). The S1 band in fezf1-disrupted group is stronger.

**Q44 :Fig. 4G: In the absence of any functional data at this point, the sox2+ ACs should be labeled according to their morphological characteristic (e.g. S1 and S4 stratifying), not a presumed functional property, which may not correlate well with the anatomical properties anyhow, according to the data shown in supplemental Fig. S5.**
Answer: Thanks for the comment. Following the reviewer’s suggestion, we have re-named AC subtypes according to their anatomical characteristic (ON-laminae and OFF-laminae) in the revised manuscript of Fig 4.

**Q45**: **Fig. 5B: How were response latencies measured? A 4s latency seems very long for a cell that is tasked with rapidly signaling direction and supporting OKR responses. Is this the true latency of functional responses, or is it limited somehow by the recording methods?**
Answer: Thanks for the comment. We agreed with the point that the reviewer raised (a latency of 4 seconds is a bit longer for a cell responsible for direction detection). This prolonged latency is probably due to the definition of the response latency used in our previous analysis, in which the response latency was defined by the time period starting at the emergence of visual stimuli on the screen (not the time the stimuli actually hit the receptive field of neurons) and ending at the time point of peak responses. In other words, the response latency we calculated in the previous version, which included the time duration between the emergence of moving bars on the screen and the bars actually touch the neurons’ dendrites, was expected to be longer than the true latency of functional responses. Due to the technical difficulties, so far, we are unable to calculate the true latency of functional responses to the moving bars. Thus, to avoid the confusion, we removed this part from the revised manuscript. Instead, we re-examined the true response latency of two AC types using the full-field ON visual stimuli (3 secs light on visual stimuli) as previously described [25]. The result showed that the response latency was 1.64 ± 0.82 secs and 1.75 ± 0.80 secs for *sox2*+ ROIs and *bhlhe22*+ ROIs, respectively. It was updated in Fig S5D and Lines 264-267 in revised manuscript.

**Q46: Fig. 5: Since the saccade frequency was significantly lower, but the saccade amplitude was unchanged in the affected animals, one might expect the eyes to reach the limit of their motion and remain pegged there during prolonged optokinetic stimulation.  Was this observed?**
Answer: Thanks for the comment.

Indeed, we observed that in the affected animals, when the eyes of larvae reached the limit of the motion, the eyes were pegged at the position for a while as the control group. The Movie S4-S7 is shown in support information.

**Q47**: **Line 349: Please quote bar widths either in degrees of visual angle or microns on the retina.**
Answer: Thanks for the comment.

Moving bar size in small range (bar width: 2,5, and 10 pixels, visual angle: 1°/pixel at visual distance of 0.36 cm) and big range (bar width: 10, 50, and 250 pixels, visual angle: 1°/pixel at visual distance of 0.36 cm) in full field of screen were applied. We have updated this in revised Materials and Methods Line 116-122 in support information.

**Q48: Fig. 6I-N: The DSI's in this dataset are lower than shown earlier. Indeed, many units show DSIs that are lower than the criterion value of 0.1 used earlier. Judging from the collected data in panels M and N, the representative traces and polar plots in panels I and K look to be extreme outliers (many SD's above the mean) rather than representative. It would be more realistic to show examples that were closer to the means of the groups.**

Answer: Thanks for the comment.

The DSI was calculated in an updated new method. Although the threshold of DS tuning was variously set in previous studies, from 0.3 to 0.5 [7, 11-16] , we set the DS tuning threshold to 0.5 in this updated revision. In this revised manuscript, all the response traces of direction-selective cells in the revised Fig. S7C and S7D were updated in the revised manuscript.

**Minor comments:**

Fig. 4A: The scale bars in the two panels are not equal in length indicating slightly different magnifications. If magnification is not preserved for comparison of the cell sizes, then why not enlarge the sox2+ cell so that it can be seen better?
Answer: Thanks for the comment. It has been updated in the revision manuscript.

Check verb tense throughout the manuscript. For example, past tense is often used to refer to immutable characteristics which is not really accurate.

Lines 69 - 83: This final paragraph seems superfluous as it simply summarizes the results.
**Answer:** Thanks for the comment. It has been updated in the revision manuscript in Line 81-84.

Line 75, "Consistently, the optokinetic…" This sentence needs to be reworded.
Answer: Thanks for the comment. It has been updated in the revision manuscript in Line 75-77.


Fig. 5C,E: although the scale bar is shown, it would be nice to know what the actual DSI’s for these examples were, as is shown in Fig. 6J,L.
Answer: Thanks for the comment. It has been updated in the revision manuscript in the Fig 5E and 5I.

Fig. 6I,K: What were the bar widths used in these examples?
Answer: Thanks for the comment. It has been updated in the revision manuscript. Response of all direction selective ACs in all bar widths were updated in Fig S7C and S7D.
References

1.	Hanson L, Ravi-Chander P, Berson D, Awatramani GB. Hierarchical retinal computations rely on hybrid chemical-electrical signaling. Cell Rep. 2023;42(2):112030. Epub 2023/01/26. doi: 10.1016/j.celrep.2023.112030. PubMed PMID: 36696265.

2.	Jain V, Murphy-Baum BL, deRosenroll G, Sethuramanujam S, Delsey M, Delaney KR, et al. The functional organization of excitation and inhibition in the dendrites of mouse direction-selective ganglion cells. Elife. 2020;9. Epub 2020/02/26. doi: 10.7554/eLife.52949. PubMed PMID: 32096758; PubMed Central PMCID: PMCPMC7069718.

3.	Euler T, Detwiler PB, Denk W. Directionally selective calcium signals in dendrites of starburst amacrine cells. Nature. 2002;418(6900):845-52. doi: 10.1038/nature00931.

4.	Chapot CA, Behrens C, Rogerson LE, Baden T, Pop S, Berens P, et al. Local Signals in Mouse Horizontal Cell Dendrites. Curr Biol. 2017;27(23):3603-15 e5. Epub 2017/11/28. doi: 10.1016/j.cub.2017.10.050. PubMed PMID: 29174891.

5.	Voigts J, Harnett MT. Somatic and Dendritic Encoding of Spatial Variables in Retrosplenial Cortex Differs during 2D Navigation. Neuron. 2020;105(2):237-45 e4. Epub 2019/11/25. doi: 10.1016/j.neuron.2019.10.016. PubMed PMID: 31759808; PubMed Central PMCID: PMCPMC6981016.

6.	Dehmelt FA, von Daranyi A, Leyden C, Arrenberg AB. Evoking and tracking zebrafish eye movement in multiple larvae with ZebEyeTrack. Nat Protoc. 2018;13(7):1539-68. Epub 2018/07/11. doi: 10.1038/s41596-018-0002-0. PubMed PMID: 29988103.

7.	Antinucci P, Suleyman O, Monfries C, Hindges R. Neural Mechanisms Generating Orientation Selectivity in the Retina. Curr Biol. 2016;26(14):1802-15. Epub 2016/07/05. doi: 10.1016/j.cub.2016.05.035. PubMed PMID: 27374343; PubMed Central PMCID: PMCPMC4963213.

8.	Peters BN, Masland RH. Responses to light of starburst amacrine cells. J Neurophysiol. 1996;75(1):469-80. Epub 1996/01/01. doi: 10.1152/jn.1996.75.1.469. PubMed PMID: 8822571.

9.	Guy J, Möck M, Staiger JF. Direction selectivity of inhibitory interneurons in mouse barrel cortex differs between interneuron subtypes. (2211-1247 (Electronic)).

10.	Pan ZH, Slaughter MM. Control of retinal information coding by GABAB receptors. J Neurosci. 1991;11(6):1810-21. Epub 1991/06/01. doi: 10.1523/JNEUROSCI.11-06-01810.1991. PubMed PMID: 1646300; PubMed Central PMCID: PMCPMC6575414.

11.	Chen M, Weng S, Deng Q, Xu Z, He S. Physiological properties of direction-selective ganglion cells in early postnatal and adult mouse retina. J Physiol. 2009;587(Pt 4):819-28. Epub 2008/12/24. doi: 10.1113/jphysiol.2008.161240. PubMed PMID: 19103682; PubMed Central PMCID: PMCPMC2669973.

12.	Wilson DE, Scholl B, Fitzpatrick D. Differential tuning of excitation and inhibition shapes direction selectivity in ferret visual cortex. Nature. 2018;560(7716):97-101. Epub 2018/07/27. doi: 10.1038/s41586-018-0354-1. PubMed PMID: 30046106; PubMed Central PMCID: PMCPMC6946183.

13.	Kim T, Freeman RD. Direction selectivity of neurons in the visual cortex is non-linear and lamina-dependent. Eur J Neurosci. 2016;43(10):1389-99. Epub 2016/03/02. doi: 10.1111/ejn.13223. PubMed PMID: 26929101; PubMed Central PMCID: PMCPMC4874909.

14.	Sato H, Katsuyama N, Tamura H, Hata Y, Tsumoto T. Mechanisms underlying direction selectivity of neurons in the primary visual cortex of the macaque. J Neurophysiol. 1995;74(4):1382-94. Epub 1995/10/01. doi: 10.1152/jn.1995.74.4.1382. PubMed PMID: 8989379.

15.	Scannell JW, Sengpiel F, Tovee MJ, Benson PJ, Blakemore C, Young MP. Visual motion processing in the anterior ectosylvian sulcus of the cat. J Neurophysiol. 1996;76(2):895-907. Epub 1996/08/01. doi: 10.1152/jn.1996.76.2.895. PubMed PMID: 8871207.

16.	Goltstein PM, Coffey EB, Roelfsema PR, Pennartz CM. In vivo two-photon Ca2+ imaging reveals selective reward effects on stimulus-specific assemblies in mouse visual cortex. J Neurosci. 2013;33(28):11540-55. Epub 2013/07/12. doi: 10.1523/JNEUROSCI.1341-12.2013. PubMed PMID: 23843524; PubMed Central PMCID: PMCPMC6618686.

17.	Yao X, Cafaro J, McLaughlin AJ, Postma FR, Paul DL, Awatramani G, et al. Gap Junctions Contribute to Differential Light Adaptation across Direction-Selective Retinal Ganglion Cells. Neuron. 2018;100(1):216-28 e6. Epub 2018/09/18. doi: 10.1016/j.neuron.2018.08.021. PubMed PMID: 30220512; PubMed Central PMCID: PMCPMC6293282.

18.	Morrie RD, Feller MB. A Dense Starburst Plexus Is Critical for Generating Direction Selectivity. Curr Biol. 2018;28(8):1204-12 e5. Epub 2018/04/03. doi: 10.1016/j.cub.2018.03.001. PubMed PMID: 29606419; PubMed Central PMCID: PMCPMC5916530.

19.	Sun LO, Jiang Z, Rivlin-Etzion M, Hand R, Brady CM, Matsuoka RL, et al. On and off retinal circuit assembly by divergent molecular mechanisms. Science. 2013;342(6158):1241974. doi: 10.1126/science.1241974. PubMed PMID: 24179230; PubMed Central PMCID: PMC3863450.

20.	Chen M, Lee S, Zhou ZJ. Local synaptic integration enables ON-OFF asymmetric and layer-specific visual information processing in vGluT3 amacrine cell dendrites. Proc Natl Acad Sci U S A. 2017;114(43):11518-23. Epub 20170927. doi: 10.1073/pnas.1711622114. PubMed PMID: 28973895; PubMed Central PMCID: PMCPMC5664540.

21.	Yoshida K, Watanabe D, Ishikane H, Tachibana M, Pastan I, Nakanishi S. A key role of starburst amacrine cells in originating retinal directional selectivity and optokinetic eye movement. Neuron. 2001;30(3):771-80. PubMed PMID: 11430810.

22.	Harris SC, Dunn FA. Asymmetric retinal direction tuning predicts optokinetic eye movements across stimulus conditions. Elife. 2023;12. Epub 2023/03/18. doi: 10.7554/eLife.81780. PubMed PMID: 36930180; PubMed Central PMCID: PMCPMC10023158.

23.	Dhande OS, Stafford BK, Lim JA, Huberman AD. Contributions of Retinal Ganglion Cells to Subcortical Visual Processing and Behaviors. Annu Rev Vis Sci. 2015;1:291-328. Epub 2015/11/24. doi: 10.1146/annurev-vision-082114-035502. PubMed PMID: 28532372.

24.	Matsuda K, Kubo F. Circuit Organization Underlying Optic Flow Processing in Zebrafish. Front Neural Circuits. 2021;15:709048. Epub 2021/08/10. doi: 10.3389/fncir.2021.709048. PubMed PMID: 34366797; PubMed Central PMCID: PMCPMC8334359.

25.	Tengolics AJ, Szarka G, Ganczer A, Szabo-Meleg E, Nyitrai M, Kovacs-Oller T, et al. Response Latency Tuning by Retinal Circuits Modulates Signal Efficiency. Sci Rep. 2019;9(1):15110. Epub 2019/10/24. doi: 10.1038/s41598-019-51756-y. PubMed PMID: 31641196; PubMed Central PMCID: PMCPMC6806000.
