# Supplementary material for: Defining morphologically and genetically distinct GABAergic/cholinergic amacrine cell subtypes in the vertebrate retina
Source: PLoS Biol. 2024 Feb 16;22(2):e3002506. doi: 10.1371/journal.pbio.3002506 (PMC10914270; doi:10.1371/journal.pbio.3002506)
Supplement: Attachment — Submitted filename: Response letter.docx [file pbio.3002506.s020.docx]

**Q1:** Reviewer #2, Takeshi Yoshimatsu (note, Reviewer 2 has signed this review): In the revised manuscript, the authors addressed most of the concerns I initially had with the original version. The new manuscript has been much improved. I just have one more thing that I would like clarification on. The visual angle of the bar stimuli used in the two-photon calcium imaging is computed based on the visual distance of 0.36 cm and the bar size on the screen (line 1010 - 1011). However, it seems that, for two-photon imaging, larval fish were almost directly placed on the screen (line 1034 - 1035, 'Vertical distance from larvae lens to the screen was 0.5 mm'). **If the calcium recordings were carried out in cells located in the dorsal region of the eye, shouldn't the visual distance be ~0.5 mm?** This detail is particularly relevant because one of the paper's interesting findings is the difference in the bar size tuning between bhlhe22 and sox2 ACs. Adie from this, the experiments and data analysis are thorough, and the findings are very exciting.
**Answer:** Thanks for the question. We apologize for the confusion. Here we re-drawed the schematic to describe the visual distance we defined in our analysis. We defined AO as the visual distance, which was 0.36 cm, rather than CO (0.5 mm).

Reviewer #3: The authors have responded in diligently to the previous reviews; however, I have one remaining concern pertinent to the basic conclusions. In my previous review I asked whether control data showing the magnitude of direction-selectivity indices (DSI's) recorded from non-directional cells under the same conditions was available. My interest was to see the variance and mean of DSI's in other non-DS retinal neurons, which could provide a basis for assessing the relative strength of DS responses in the two novel ACs. However, in this revised manuscript, responsive sox2+ and bhlhe22+ ACs were each divided in two groups; DS and non-DS cells. Surprisingly, the DS cells represent only a minor fraction of the total sample for each cell-type (Figs 5 & 6).

These directional sox2+ and bhlhe22+ ACs are compared to the directional GABAergic/cholinergic starburst ACs reported in mammalian retinas. Starburst ACs uniformly show robust directional responses, so it is perhaps surprising that most of the novel sox2+ and bhlhe22+ ACs are non-directional. Thus, it would be more accurate to conclude from the data that the sox2+ and bhlhe22+ AC types in zebra fish are not direction selective, unlike their presumed mammalian counterparts. Consistent with this conclusion are the very modest effects that cell-specific KO of the ACs have on the OKR (Fig. 5).

The description of these novel ACs and their homology with mammalian AC types is interesting and significant. The study nicely documents functionally, morphologically, and genetically distinct GABAergic/cholinergic amacrine cells in a lower vertebrate retina. However, the evidence doesn't support the claim that they are functionally homologous to the mammalian cells or that they serve a major role in directional signaling. I would recommend that the authors revise the title, abstract, and discussion to reflect the findings more accurately.

COMMENTS FROM THE ACADEMIC EDITOR
Judging from the data provided by the authors, I am pretty sure their assertion of DS cannot be substantiated from these traces. In almost no case, the multiple repeats to the same stimulus give the same responses, and the "DS preference" only results from considering the average of some spontaneous events. Imagine a cell responds poorly to a given stimulus, but there is one or two spontaneous events that roughly coincide with when a stimulus was played. Then, on average, this cell would come out as directionally selective. However, these types of spontaneous events should have been filtered out. People typically compute some sort of quality metric for the repeatability of responses. I do not see such a thing used, and certainly, if used properly, it would kick out pretty much all of the trials shown in the pdf. Also, some of the "responses" look like motion artifacts (their kinetics are not plausibly explained by neural processing or calcium dynamics).

Therefore, based on the data I can see, I am with the reviewer: Either, the cells are not DS, or their DS-ness remains to be established with additional experiments and analysis.

In light of that I would strongly suggest the authors focus on the very nice description of these cells presence, organisation, genetics etc, and - importantly, the provision of the exceptionally useful transgenic lines that go with them, but keep the functional data very cautious, probably concluding that for now there is no strong evidence that there is DS, but importantly, that this doesnt mean that it is not there. It could also have been missed for technical reasons, and future work will be needed to carefully check this.

**Answer:**

Thanks for the comments raised by the reviewer and the editor. In this round of revision, we have updated some ROIs that were originally generated by the automatic method because they were found to be mistakenly assigned to the regions without any CAMP6 signals, which is probably due to technical caveats of the automatic method. We then re-analyzed all responses based on these updated ROIs. Although we still found a fraction of either AC type (7.8% of *bhlhe22*+ ACs and 28.8% of *sox2*+ ACs) with the directional selectivity property, either AC type showed the reproducible DS responses (Figs 5D,5H,S5A,S5B). We have uploaded two documents (sox2_DS_rois_traces_album.pdf and bhlhe22_DS_rois_traces_album.pdf) containing all updated DS rois. Together with the findings of the reduction of OKR by ablating either AC type, our data still suggested that both AC types showed directional selectivity. However, we also agreed with the comments from the reviewer and the editor that two AC types of the zebrafish retina showed a difference from their mammalian counterparts in terms of the proportion of cells with DS responses, which need to be further determined in the future. Therefore, in the revised manuscript, we have updated the title, the abstract, and the result and discussion related to the conclusion regarding the DS properties of two AC types. We hope these updates will improve the accuracy of our findings and are satisfying to the reviewer and the editor.
